# A Reversible Zinc-Ion Microbattery from a Printed Gel-Electrolyte and a Carbon–Zinc Formulation

**DOI:** 10.1021/acsami.5c05599

**Published:** 2025-06-23

**Authors:** Stefano Tagliaferri, Nagaraju Goli, Maria S. Sokolikova, Haoyu Bai, Caiwu Liang, Ifan E. L. Stephens, Cecilia Mattevi

**Affiliations:** Department of Materials, 4615 Imperial College London, London SW7 2AZ, United Kingdom

**Keywords:** Printed Zn-ion battery, dendrite-free anode, gel-electrolyte, MnO_2_ cathode, capacity, proximity sensor

## Abstract

Aqueous zinc ion batteries (ZIBs) are attracting increasing attention due to their low cost, earth abundance, and safety. So far, they have been regarded as a promising battery system for large scale grid applications, while here, we demonstrate prospects of their use to power portable devices. We report the fabrication of a rechargeable ZIB with interdigitated geometry capable of powering a commercial sensor for days. A full battery was assembled using aqueous and scalable formulations with a printed anode based on zinc powder and carbon black, a printed colloidal electrolyte, and a printed MnO_2_ cathode. The anode withstands more than 500 h of galvanostatic plating/stripping with a low overpotential of ∼32.2 mV, and the ZIB displays a capacity of ∼1.3 mAh/cm^2^ (∼129 mAh/g) and retains ∼66% of its capacity after 100 cycles. Finally, we show how this battery can power a Bluetooth proximity sensor, providing a voltage of 3.2 V for more than 3 days of continuous operation.

## Graphical abstract

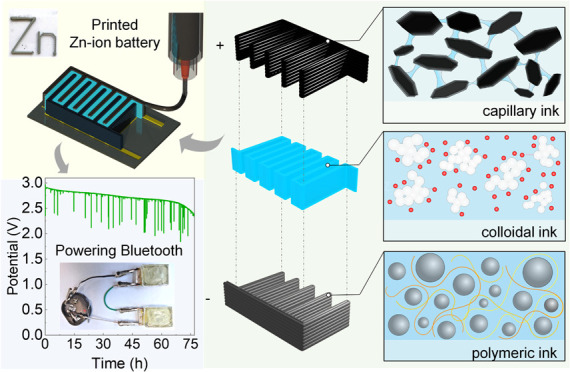

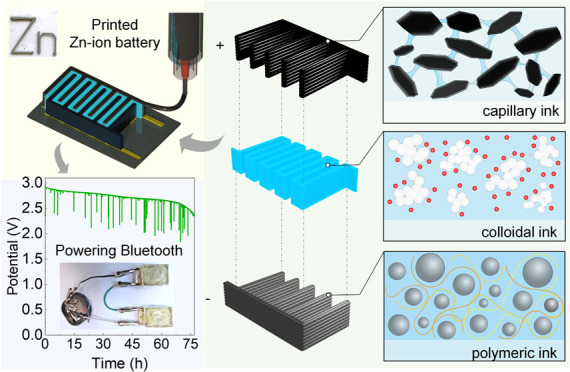

## Introduction

Batteries to power the increasingly ubiquitous wearable electronic devices ought to be safe, rechargeable, durable, conformable, made of environmentally friendly materials, and manufactured through material-efficient processes with low carbon footprint.^1–4^ Zinc-ion batteries (ZIBs) can be viewed as sustainable batteries, owing to their compatibility with aqueous electrolytes and their smaller cradle-to-gate environmental impact in terms of CO_2_ emissions per kWh of energy stored.^5–7^ Zinc is an abundant resource, presenting a high theoretical capacity (∼820 mAh/g) and a low redox potential (−0.76 V vs the standard hydrogen electrode) as a negative battery electrode.^8–10^ However, the majority of zinc batteries on the market today are nonrechargeable (i.e., primary batteries); thus, they are discarded after use, contributing to the growing stream of electronic waste.^11–14^ Additionally, commercial zinc batteries are available in a restricted variety of shapes and form factors, which hinder possible integration into wearable devices.^15^

Extrusion based 3D printing processes from viscoelastic inks meet the demand for shape customization and sustainable manufacturing of batteries, especially when water-based ink formulations and room-temperature deposition are employed.^16,17^ Additionally, they enable one to finely engineer the electrode composition through the formulation of inks and to structure the electrode over small footprint areas.^18^ Significant technological challenges,^19,20^ such as ink formulation, postprocessing, and cycling stability (zinc dendritic formation, hydrogen evaluation issues, etc.),^17^ have hindered the demonstration of printed ZIB at the full-cell level. The composition of the inks for 3D printing must be tuned to fabricate structures with high shape retention and spatial resolution, which also present adequate functional properties for battery cathodes, anodes, and electrolytes, respectively.^21–23^ To this end, electrochemically inactive additives and solvents are included in the ink formulation and then removed after the printing process.^24–26^ Most of the research on 3D printed zinc batteries so far has investigated the fabrication of a single battery component (mainly printing a zinc anode), which utilizes large volumes of organic solvent with a standard electrode geometry.^27–29^

In this work, we demonstrate a fully printed rechargeable ZIB with an interdigitated geometry capable of powering commercial sensors for over 3 days (Scheme 1). The electrodes and electrolyte are patterned using direct-ink-writing of water-based inks (DIW) at room-temperature.^30^ This DIW relies solely on the ink rheology to obtain self-standing 3D structures with complex geometric features (Scheme 1b). The 3D printed zinc anodes are based on zinc powder; they are mechanically stable and able to withstand more than 500 h of galvanostatic plating/stripping, preserving a low deposition overpotential of ∼32.2 mV at 1 mA cm^–2^. The printed zinc anodes were coupled with manganese oxide cathodes, achieving a capacity of ∼1.3 mA cm^–2^ in a full cell configuration. A thixotropic electrolyte ink was designed with the aid of Computational Fluid Dynamics (CFD) simulations. It presented high ionic conductivity (∼6 S m^–1^) and was used to fabricate an all-printed interdigitated battery (Scheme 1c). This exhibited an operating voltage of 1.8 V and could power a Bluetooth proximity sensor for over 3 days. This work demonstrates the potential of printed batteries with freeform design and miniaturized dimensions to power wearable sensors, and more widely, it shows their prospects to serve for the Internet-of-Things.

**Scheme 1:**
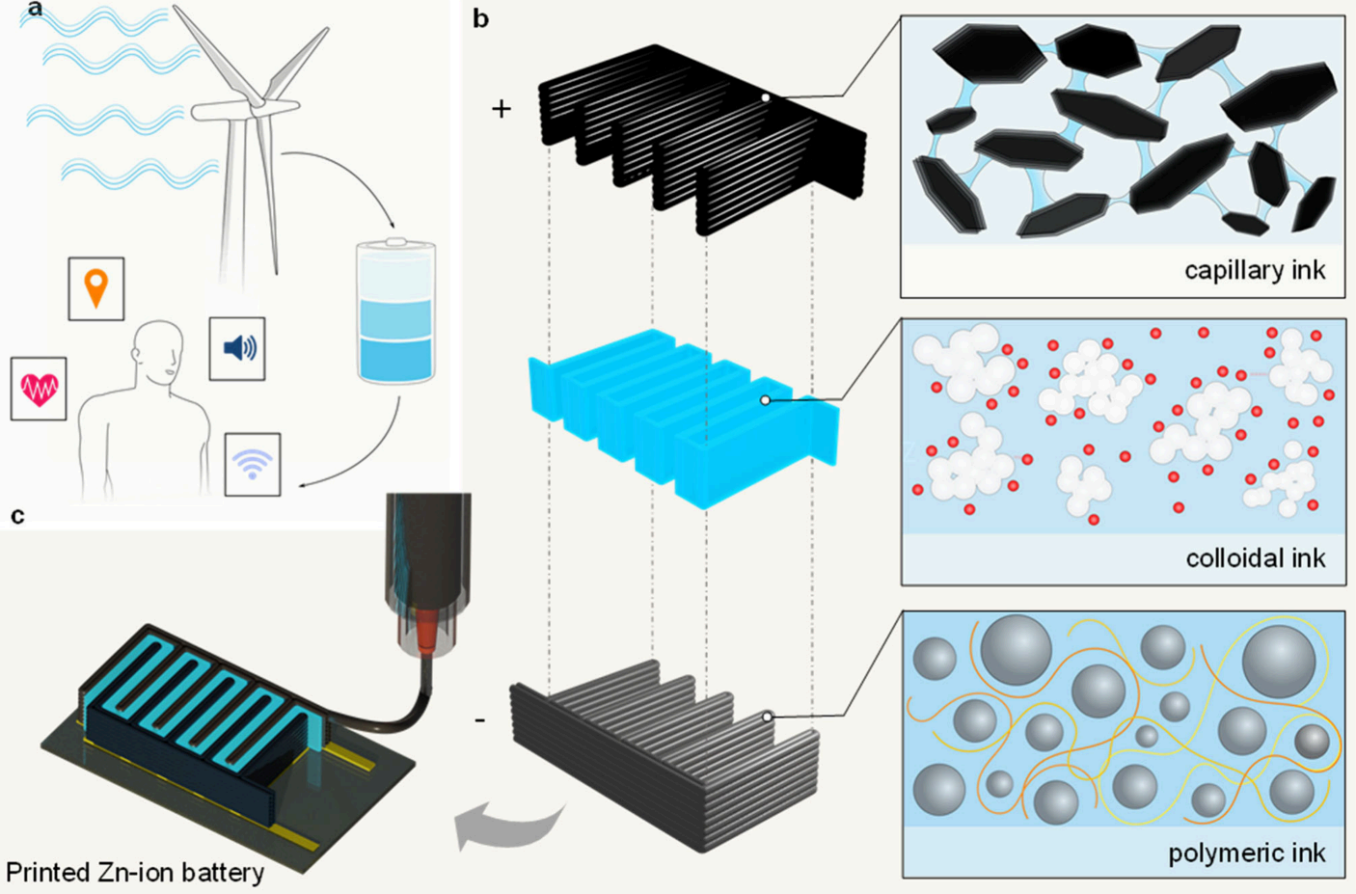
Details of the Fully Printed Rechargeable ZIB and its Applications^a^

a (a) Schematic presentation showing different applications of ZIBs, including renewable energy storage, wearable devices, and smart home systems.^31^ (b) Schematic illustration of the formulated aqueous inks used for the 3D printing of ZIB in this work: capillary ink, colloidal ink, and polymeric ink, respectively. (c) Demonstration of the 3D printing process for the fabrication of ZIB, showcasing the layering technique used to build the microbattery electrode architectures.

## Results and Discussion

Micron-sized zinc spheres were used as the principal active material for the anode ink (Figure S1a). Dense suspension of non-Brownian (>1 μm) particles can form yield stress fluids at sufficiently high volume fractions;^13^ however, polymeric additives are required to stabilize the suspension and bind the non-Brownian zinc particles together. Here, we used poly­(vinyl alcohol) as a binder, which is rich in hydrophilic hydroxyl moieties, thus displaying a swelling ratio of >300% in aqueous electrolytes (Figure S1c). The ability of the binder to swell in the electrolyte is crucial for 3D electrodes to ensure the access of ions to the bulk of the structure, lowering the electrochemical impedance (Figure S1d). The SEM images of printed zinc electrodes demonstrate woodpile architecture electrodes with three-dimensional (3D) shapes (Figure 1b). Micron-sized zinc particles were blended with carbon black at different concentrations, as included in Figure 1c. After drying at 90 °C, the binder undergoes partial crystallization,^32^ becoming stable in water. At a concentration above the critical packing fraction for zinc particles, the zinc ink can form load-bearing filaments, presenting a yield stress of ∼170 Pa (Figure 1d). The high yield stress and the elastic behavior of the ink at rest (*G*′ ∼ 2 × 10^5^ Pa, Figure 1d and Figure S2) enable the fabrication of space-spanning struts, thin walls, and overhangs, demonstrating a great degree of geometric freedom for the fabrication of three-dimensional electrodes (Figure 1a). However, the naturally occurring oxide layer on zinc particles might prevent long-range electron transport in electrodes that are several millimeters thick; hence, conductive CB was included in the ink formulation (Figure 1c, Figure S1b,c). The amount of conductive carbon was progressively increased until the difference between the conductivity of the initial oxidized state and the unoxidized state, i.e., after soaking in a depassivating acidic solution (pH ∼ 2), became negligible (∼23 S m^–1^), indicating that the carbon formed a percolating network able to quickly deliver electrons to all zinc particles inside the 3D electrodes (Figure 1f). X-ray Computed Tomography (XRT) and SEM imaging demonstrate that the zinc particles are uniformly dispersed within the printed filaments alongside carbon black particles (Figure 1e).

**Figure 1:**
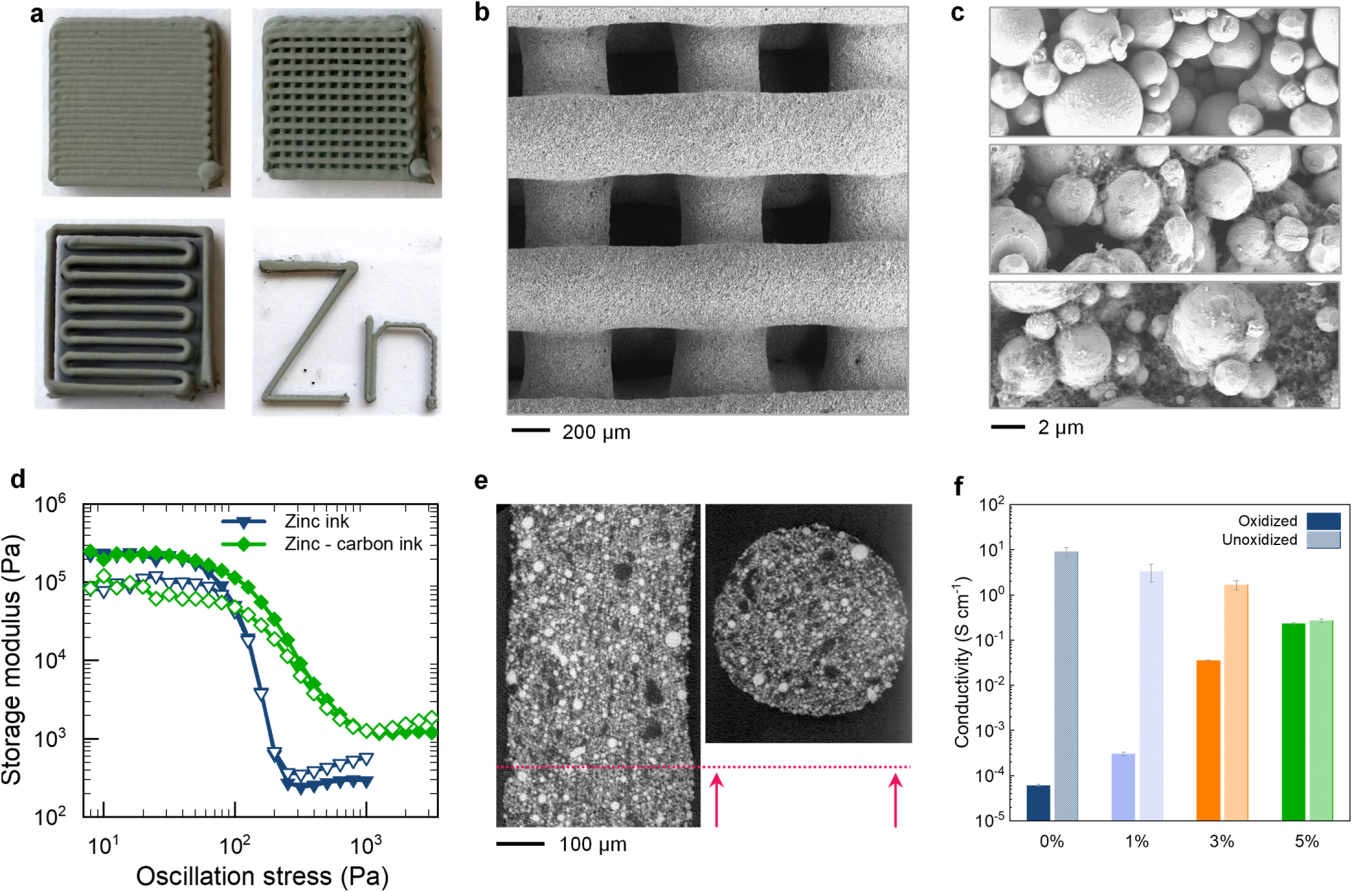
(a) Photographic images of the 3D printed structures fabricated using zinc inks. (b) SEM image of the woodpile electrode printed using the zinc-CB 5% ink. (c) High-magnification SEM images of printed electrodes at increasing content of carbon black: 0% (top), 1% (middle), and 5% (bottom). (d) Oscillatory amplitude sweep tests for the pristine zinc and zinc-CB 5% inks. (e) X-ray tomographic slice of the zinc-CB 5% and (f) comparison of the electrical conductivity of zinc structures at different concentrations of carbon black before and after acid treatment; corresponding *I*–*V* plots of the printed electrodes are included in Figure S1.

The stability of the printed zinc electrodes in aqueous electrolytes was initially investigated in symmetric cells via galvanostatic charge–discharge tests, simulating the cyclic dissolution and deposition of zinc during the battery operation.^33^ Zinc anodes with a higher content of conductive carbon (5%) present a smaller deposition overpotential (∼50 and 100 mV at 1 and 10 mA/cm^2^), attributable to the improved conductivity of these structures, which ensures faster electron transport and larger electrochemically active regions (Figure 2a and Figure S5). The electrodes without carbon are affected by large voltage spikes (>500 mV)^8^ during cycling at 10 mA cm^–2^, likely due to the formation of dead zinc, which disrupts charge transfer (Figure S5c).^34,35^ Conversely, all of the electrodes containing carbon retain a stable overpotential for more than 500 h of continuous cycling, without displaying sudden variations in polarization resulting from increased charge transfer resistance or dendritic growth (Figure 2b-(i)). The cell with the highest carbon content exhibits the lowest deposition overpotential (∼32.2 mV; Figure 2b-(ii)) and the highest Coulombic efficiency (∼95%, Figure 2d). The latter was measured through the half-cell reservoir method,^36,37^ indicating that only a small fraction of the zinc is lost in irreversible side reactions (Figures S4 and S5b).^38^ In Figure 2b, there is a decrease of the polarization–voltage with increasing CB content, owing to improved electron transport and particle distribution homogeneity. The 3D zinc electrodes are mechanically stable, showing progressive densification with no lateral buckling or critical failure under a compression load of ∼8 MPa (Figure S3). Remarkably, the printed electrodes can preserve their shape and mechanical stability even after 5 h of continuous discharge at 5 mA cm^–2^. Although partial dissolution of the electrode is evident from *ex situ* SEM images (increase in porosity in Figure 2f), the reduction in cross sectional area is not so severe to affect the load bearing capacity of the printed struts, as illustrated from SEM and XCT after discharge (Figure 2f,g). The surface of the electrodes appears covered by a thin layer of nanoplatelets after 500 h of charge–discharge, identified as zinc hydroxysulfates from X-ray diffraction (Figure 2h). The hexagonal hydroxysulfates originate from the progressive corrosion of zinc, which determines an increase of pH in the proximity of the electrode surface. However, the hydroxysulfates do not form a compact layer that blocks charge transport inside the electrode, as demonstrated by impedance spectroscopy (Figure 2e and Figure S6). The kinetic of the zinc corrosion was further investigated via linear sweep voltammetry (LSV) and *in situ* electrochemical mass spectrometry.^39,40^ The Tafel analysis (Figure S7a,b) shows that the CB 5% anode displays a corrosion current of ∼731 μA/cm^2^ with a corrosion rate of ∼3.8 nmol/s·cm^2^, which means the electrode is progressively dissolving in an acidic aqueous environment. At the open circuit potential, the CB 5% electrode produces ∼0.14 nmol/s/cm^2^ of hydrogen in stationary conditions (Figure 2i), which is much lower than the corrosion rate estimated from the Tafel analysis. Furthermore, the rate of hydrogen evolution marginally changed during the charge–discharge experiments (Figure S7c). Both the stripping/plating experiments and *in situ* mass spectrometry indicate that printed zinc electrodes are stable over 500 h, despite the formation of a thin layer of zinc hydroxy sulfate, since its porosity does not block the charge transfer processes from the inner part of the struts that form the electrodes.

**Figure 2:**
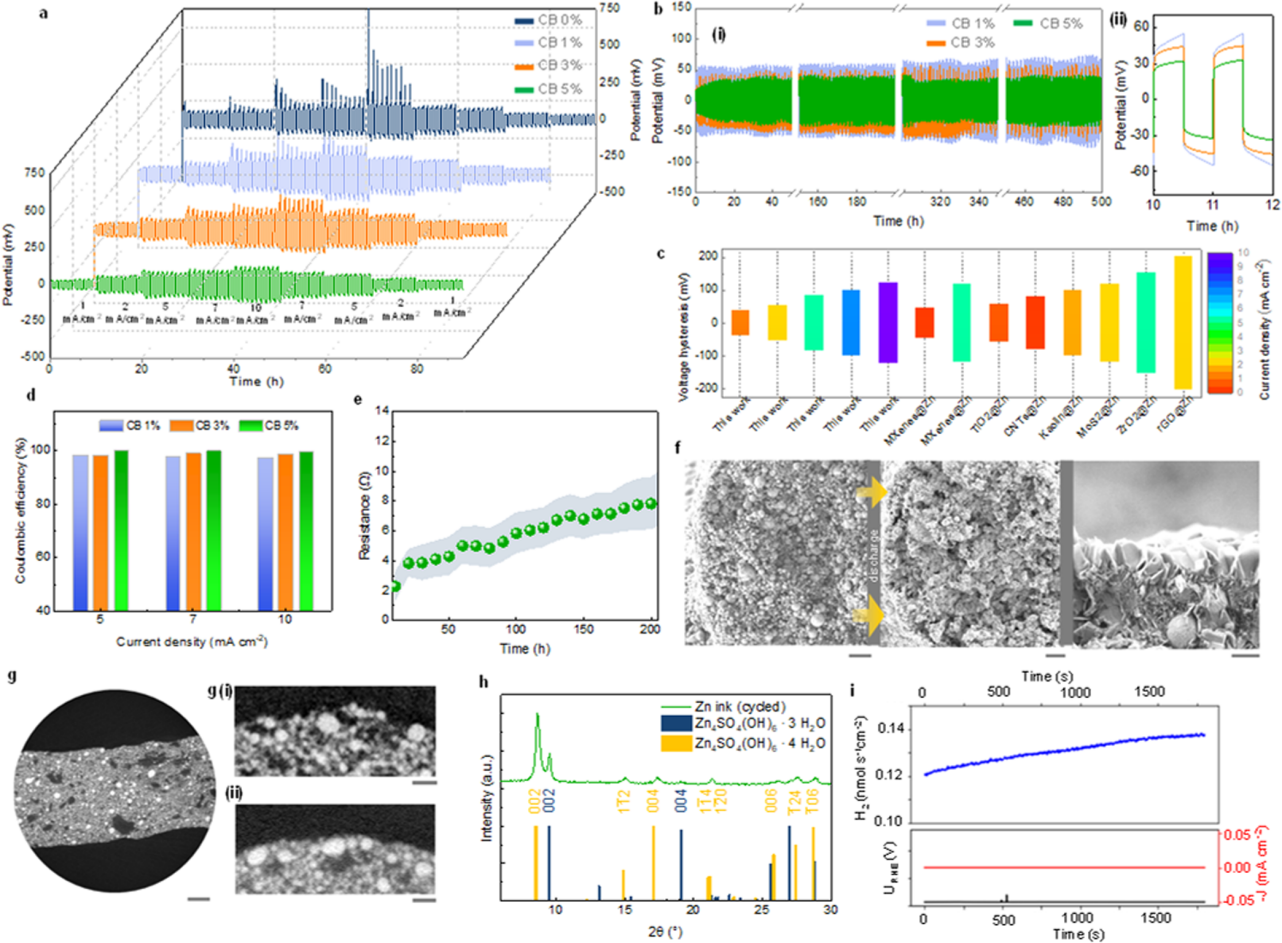
(a) Comparison of the plating-stripping overpotential of the 3D printed zinc-CB-based symmetric cells at increasing current density values, (b) cycling stability of the symmetric cells during plating-stripping tests at 1 mA cm^–2^ for 500 h (ii is a zoomed-in section of (b, i)). (c) Comparison of the voltage hysteresis for the plating and stripping of zinc-CB electrodes with previously reported zinc anodes (reference is included in Table S1). (d) Coulombic efficiencies of the 3D printed electrodes with different carbon black contents. (e) Variation in the resistances (extracted from in situ EIS) over plating/stripping cycling for the symmetric 3D printed zinc-CB5% electrodes. (f) SEM images of printed electrode (5% CB ink) before (left) and after (middle) continuous discharge for 5 h at 5 mA cm^–2^ and after 500 h (right) of galvanostatic cycling at 1 mA cm^–2^ (scale bars: 20 μm). (g) X-ray tomographic slice of a 3D printed zinc electrode (5% CB) after 500 h of plating/stripping cycles (scale bar: 100 μm); magnified tomographic slices at the edge part of the printed electrode comparing the surface of an uncycled electrode (g)­(i) and a cycled electrode (g)­(ii) (scale bars: 20 μm). (h) Ex situ XRD on the zinc anode after the cycling process and (i) in situ mass spectroscopy analysis of the zinc-CB electrode.

The cathode consisted of a core printed graphene structured overlaid by a thin layer of MnO_2_. A capillary suspension of graphene nanoplatelets in water was 3D printed to fabricate highly conductive scaffolds.^41^ Subsequently, MnO_2_ was grown on the 3D graphene (MnO_2_-graphene) scaffolds via electrochemical deposition. The electrodeposited manganese oxide forms a uniform layer of thin nanosheets that completely cover the printed graphene electrodes (Figure 3a,b and Figure S8). X-ray photoelectron spectroscopy (XPS) confirms the formation of MnO_2_, as indicated by the binding energy of the Mn 2p_3/2_ and Mn 2p_5/2_ peaks at ∼642.6 and 654.8 eV, respectively,^42^ which are characteristic of a +4 oxidation state (Figure 3c). A 3D printable gel-electrolyte was formulated using a colloidal dispersion of fumed silica in water (5 wt %) with 2 M ZnSO_4_ (Figure 3d,e and Figures S9 and S10). The gel electrolyte behaves as a solid material with a predominantly elastic response at rest (*G*_eq_′ ∼ 5.2 × 10^3^ Pa vs *G*_eq_″ ∼ 70 Pa) and a shear thinning behavior, as indicated by its oscillatory frequency response at low strain amplitudes and flow behavior rheological analysis (Figure S9). To optimize the design effectiveness of the gel, we performed computational fluid dynamics (CFD) simulations to understand the behavior of the colloidal electrolyte under flow conditions. CFD simulations (Figure 3f) show the velocity profile of the ink inside the printing cartridge, which is negligible, and reveal that the ink experiences the highest shear in the inner part of the printing nozzle. The shear rate (γ̇) was further quantified from the velocity profile in the nozzle, which shows that the central region of the extrudate (resenting a diameter ∼200 μm) experiences a small shear rate upon extrusion <10 s^–1^. In contrast, γ̇ reaches a maximum value of ∼280 s^–1^ in the sheared region near the nozzle wall. The recovery of the ink viscoelastic properties after shear (at 280 s^–1^) was investigated with three-interval thixotropy tests (3ITTs, Figure 3g). 3ITTs demonstrate that the outer region of the ink behaves as a mechanically weak gel during the first few minutes after extrusion, but the lower storage modulus can promote the adhesion and infiltration of the ink into the printed electrodes. At the same time, the presence of an unyielded core can support the electrolyte structure, preventing leakage outside of the footprint area of the device. Moreover, the gel-electrolyte also exhibits a high ionic conductivity of 6 S/m, which is significantly higher than other gel electrolytes reported for ZIBs (Figure 3f, Figure S10b,c). Noteworthy, the ionic conductivity of the colloidal gel-electrolyte is close to that of conventional liquid ZnSO_4_ electrolytes (inset: Figure S10b). Accordingly, the gel electrolyte was tested in a symmetric Zn cell configuration, which ensured stable Zn plating/stripping cycles for more than ∼360 h (Figure S10e). Furthermore, the gel-electrolyte exhibits an excellent Coulombic efficiency of ∼97% and ∼99% at a plating-stripping current density of 2 and 10 mA/cm^2^ respectively (Figure S10d).

**Figure 3:**
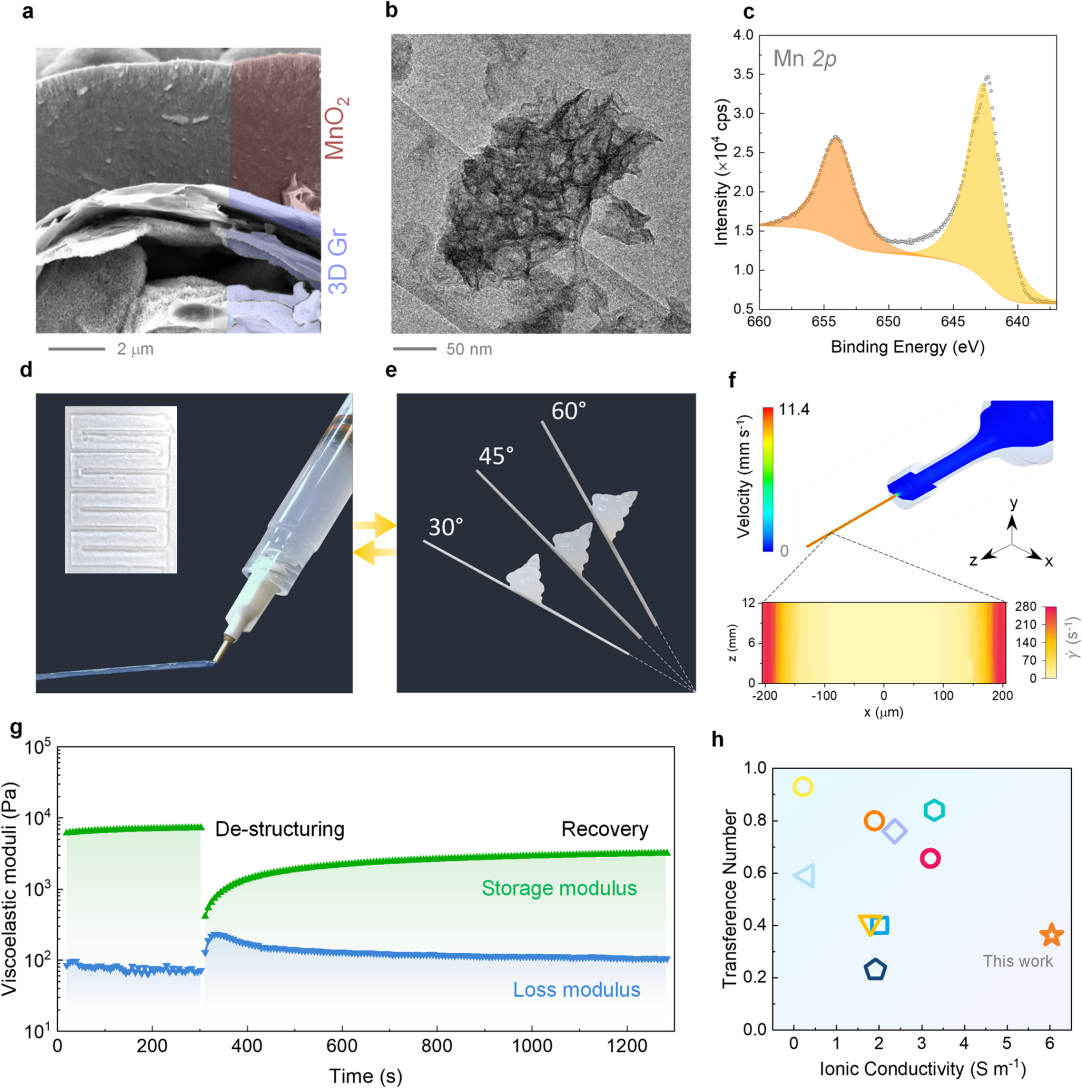
(a) SEM image of the electrochemically deposited MnO_2_ on printed conductive graphene scaffold; (b) TEM image of the MnO_2_ nanosheet; (c) XPS analysis of the Mn 2p_1/2_ and 2p_3/2_ core levels from MnO_2_. (d) 3D printed gel electrolyte; (e) self-standing gel electrolyte on glass with different angles; (f) velocity profile of the gel electrolyte in the extrusion cartridge and nozzle determined with CFD simulations (extrusion speed 6 mm s^–1^); the magnified portion from the nozzle showing the shear rate profile inside the printing nozzle. (g) 3ITTs on the gel electrolyte after a continuous rotational shear at 280 s^–1^ (the corresponding region was highlighted in green). (h) Comparison of the transference number and ionic conductivity of the gel electrolyte with the previously reported zinc-ion gel electrolytes (references are given in Table S2).

The 3D printed zinc-CB anode was coupled with the electrochemically deposited MnO_2_-graphene cathode to fabricate Zn-ion microbatteries (ZIBs; details are given in Supporting Information). The cyclic voltammograms of the printed ZIBs could exhibit a nearly stable current response with two oxidation and reduction peaks at ∼1.57/1.61 and ∼1.25/1.38 V (Figure 4a and Figure S11). The stable redox peaks could arise from the conversion of MnO_2_ to ZnMn_2_O_4_ (Figure S12), from the reaction with H^+^ to form MnOOH, or from a combined mechanism.^43^ As shown in the charge–discharge curves of ZIBs (Figure 4b), the areal capacity of the full cell increases while increasing the electrodeposition time of MnO_2_ on the graphene scaffold. The cell with the growth time of 75 min achieved an initial capacity of ∼1.3 mAh/cm^2^ at 0.1 mA/cm^2^ with the best rate performance, retaining the highest capacity when tested at increasing current densities from 0.1 to 2 mA/cm^2^ (Figure 4c). The fabricated microbattery also showed a maximum gravimetric specific capacity of 129 mAh/g at a discharge current density of 0.1 mA/cm^2^ with a rate capability of 27.1% at a high discharge current density of 2 mA/cm^2^. Moreover, the cell shows a Coulombic efficiency of ∼97% with a capacity retention of ∼66% after 100 cycles (Figure 4d). The capacity of the printed cell surpasses previous reports on ZIBs (Figure 4e). Moreover, we have compared the performance of our printed battery to existing aqueous battery technologies reported in the literature. For instance, O’Dwyer et al.^44^ demonstrated a printed aqueous lithium-ion battery that achieved a cell-level capacity of 1.86 mAh, a working voltage of 1.3 V and retained 77.7% of its capacity after 50 cycles. Similarly, Wu et al.^45^ reported a fully printed flexible aqueous sodium-ion battery with a working voltage of 1.0 V, a discharge capacity of 40.1 mAh/g, and a cycling stability of 78% after 100 cycles. In our case, it was possible to enhance the capacity of the printed battery up to ∼9.5 mAh by increasing its footprint area to 6.25 cm^2^ (Figure S13). For portable electronic applications, a fully printed ZIB was fabricated via printing interdigitated zinc-CB and MnO_2_-graphene electrodes onto a prepatterned gold current collector (Figure 4f). The colloidal gel-electrolyte was printed in the gaps between the electrodes, and the cell was encapsulated with an epoxy resin (Figure 4g). The printed cell showed a similar electrochemical response like woodpile electrodes tested in a conventional electrolyte with a capacity of 0.7 mAh at 2.25 mA (Figure S14). A commercial Bluetooth proximity sensor was powered by the printed ZIBs (connected in series) to provide sufficient voltage (2–3.6 V). The voltage drop of the ZIBs was monitored during operation (Figure 4h,i), demonstrating a continuous operating time of >75 h. The spikes observed in the potential curve in Figure 4h,i are attributed to the “ringing” effect of the Bluetooth device, which occurs as it intermittently consumes power during operation. Bluetooth devices tend to have fluctuating power demands, especially when actively transmitting or receiving signals, which causes momentary voltage drops, resulting in the observed spikes in the potential curve. The spikes reflect these brief periods of increased current draw from the printed zinc-ion batteries. Once the Bluetooth module is turned off or goes into a low-power idle state, the voltage recovers to its baseline level because the power demand decreases significantly. Overall, these results show that printed ZIBs devices can be used as a sustainable alternative to traditional battery technologies with potential applications in different fields such as the Internet-of-Things, renewable energy systems, and flexible electronics.

**Figure 4:**
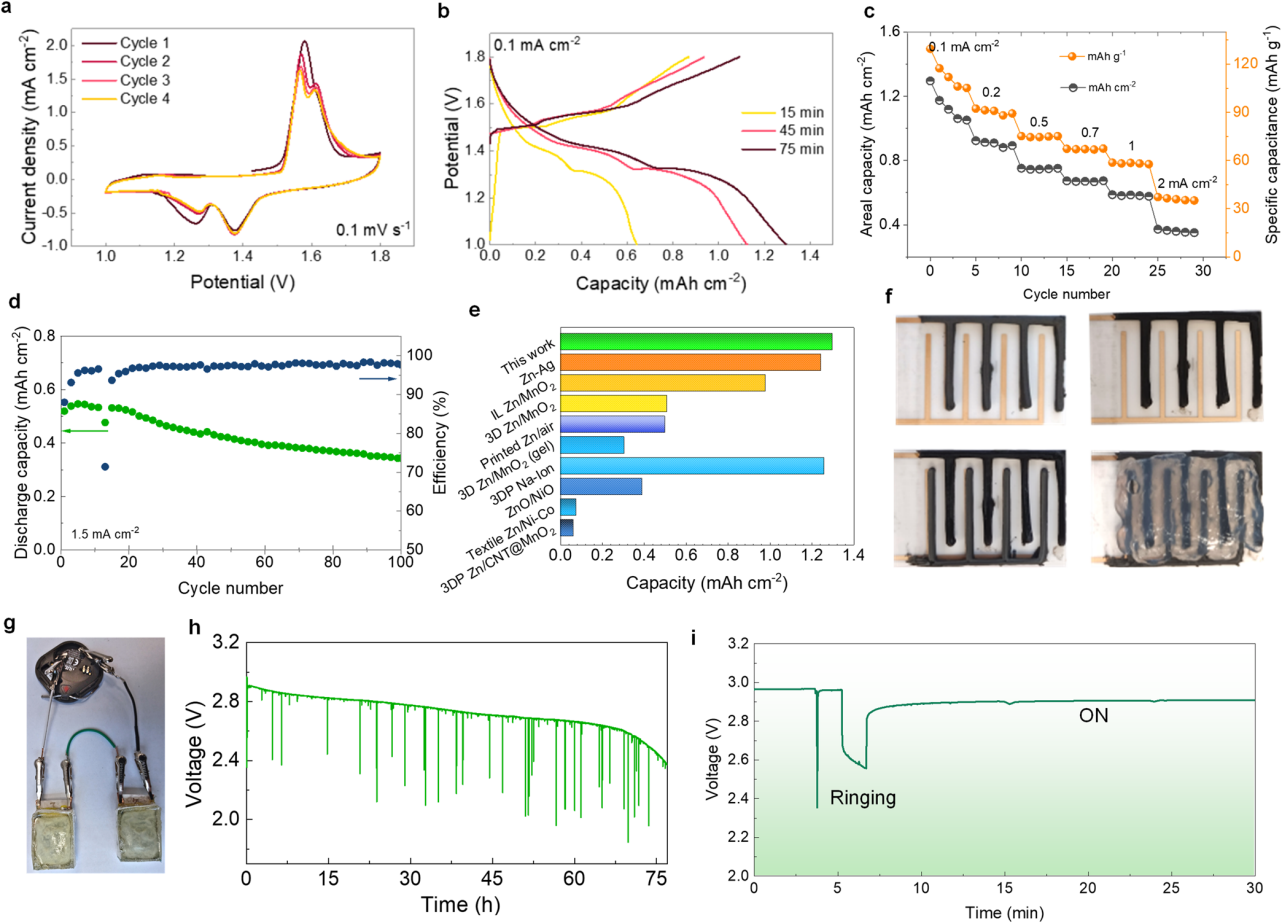
(a) CV curves of the printed woodpile-shaped ZIB (from cycles 1 to 4) at constant scan rate of 0.1 mV/s. (b) Charge–discharge curves of microbatteries assembled with MnO_2_ cathodes deposited over different times and (c) areal capacity and gravimetric capacity performance of ZIBs. (d) Cycling stability of ZIB at 1.5 mA/cm^2^; (e) comparison of areal capacity with previously reported printed batteries (references are given in Table S3). (f) Digital photographs showing the fabrication steps for the interdigitated battery: printing of the graphene electrode (top-left), electrodeposition of MnO_2_ (top-right), printing of the zinc electrode (bottom-left), and printing of the gel electrolyte (bottom right). (g) Digital photograph of two encapsulated interdigitated batteries connected to a Bluetooth proximity sensor; (h) discharge profile of the series-connected ZIBs under a constant current, demonstrating stable operation over 75 h with intermittent voltage drops due to load changes/signal transmission events, and (i) voltage response during Bluetooth activation, showing an initial voltage drop upon “ringing” (activation), followed by a stable operating potential during continuous operation.

## Conclusions

In summary, we have formulated aqueous inks of zinc spheres, carbon black, and a sustainable binder to print mechanically robust 3D anodes. They display structural integrity and electrochemical performance over extensive cycling. The integration of carbon black notably reduced the deposition overpotential to ∼32.2 mV and ensured long-term cycling (∼500 h) with a final Coulombic efficiency of ∼95%. A fully printed ZIB was designed using a newly formulated printable colloidal electrolyte and a printed MnO_2_-coated graphene cathode. Owing to the high ionic conductivity (6 S/m) of the gel-electrolyte and the cathode design, the fabricated ZIB showed a capacity of ∼1.3 mAh/cm^2^, a specific capacity of ∼129 mAh/g at 0.1 mA/cm^2^, Coulombic efficiency of ∼97%, and a capacity retention of ∼66% after 100 cycles. The assembled interdigitated ZIB demonstrated the ability to power a Bluetooth proximity sensor for more than three days of continuous operation. Overall, we have demonstrated that printed ZIBs could be considered a sustainable and efficient alternative to traditional battery technologies for powering sensors, paving the way toward the development of power sources for the Internet-of-Things and healthcare device systems.

## Experimental Details

### Formulation of Polymeric Zn-Carbon Black Anode (Zn-CB) Ink

Zinc anode inks were formulated by mixing zinc powder (<10 μm, Sigma-Aldrich) in a water-dissolvable poly­(vinyl alcohol) (10 wt %) solution (Sigma-Aldrich). The mixture was homogenized in a planetary mixer (Thinky ARE-250) for 2 min at 1800 rpm. Different amounts of acetylene carbon black (Alfa Aesar) were gradually added to the zinc slurry, and the carbon-to-zinc mass ratios were adjusted: 1:100 (1% ink), 3:100 (3% ink), and 5:100 (5% ink), respectively. After each addition of carbon black, the Zn-CB inks were homogenized again at 1800 rpm for 2 min. The final ink was cooled to room temperature and loaded in a plastic cartridge for printing.

### Preparation of Printable Colloidal Electrolyte

Zinc sulfate (ZnSO_4_)-based printable electrolyte was formulated by creating a colloidal suspension of aqueous ZnSO_4_ (Thermo Fisher Scientific) and 5 wt % of fumed silica (Sigma-Aldrich) in deionized water. The concentration of ZnSO_4_ was adjusted to 2 mol/L in the fumed silica dispersion. The suspension was homogenized in a planetary mixer at 1800 rpm for 2 min and left to rest overnight prior to 3D printing.

### Preparation of Capillary Ink

The formulation of capillary inks was obtained by dispersing 16.7 wt % of graphene nanoplatelets (5 μm, Sigma-Aldrich) in 5 wt % of sodium carboxymethylcellulose (Sigma-Aldrich) solution. Sodium carboxymethyl cellulose acted as a viscosifier for the graphene suspension, promoting the colloidal stability of the nanoplatelets. The graphene dispersion was homogenized by using a planetary mixer at 2000 rpm for 10 min. After mixing, the dispersion was cooled to room temperature and ∼2 vol % of 1-octanol (Sigma-Aldrich) was gradually added as a secondary liquid phase to form bridges between the graphene platelets. The ink was homogenized at 2000 rpm after each addition and then stored at ∼4 °C before printing.

### Rheological Characterizations

The rheological properties of the formulated inks were measured by a Discovery Hybrid HR1 rotational rheometer (TA Instruments), equipped with 40 mm stainless-steel plates. The flow properties of the inks were measured in controlled shear rate mode, applying a logarithmic shear rate ramp in the range of 0.1–100 s^–1^ and measuring the corresponding shear stress. Oscillatory tests were used to characterize the viscoelastic properties of the inks in the solid regime and the transition to the liquid regime. During frequency-sweep tests, a small-amplitude sinusoidal strain (0.1%) was applied to the sample, and the frequency of the oscillation was swept between 0.1 and 100 Hz.

### 3D Printing of Inks

The prepared inks (Zn-CB, colloidal gel-electrolyte, and capillary Gr inks) were separately loaded into the 3 mL plastic syringes, and they were connected to an extrusion-based 3D printer composed of a stainless-steel plunger. A customized extrusion-based 3D printer was used to print the prepared inks, and it consists of a gantry robot with three linear actuators that control the movement of the printing head along the *XYZ* directions. The inks were printed into different configurations with woodpile designs and interdigitated designs. The woodpile geometry presents alternating layers of equally spaced struts, forming open channels that ensure the diffusion of the electrolyte and provide high mechanical stability against lateral buckling. On the other hand, the interdigitated geometry allows the simultaneous fabrication of the negative and positive electrodes on a single substrate, simplifying the integration of printed batteries. All the inks were printed on graphite foil substrates (woodpile electrodes) and on prepatterned gold current collectors for interdigitated microbatteries. The prepared colloidal electrolyte ink was also printed on a glass substrate for ionic conductivity measurements.

### Post-treatment of Printed Electrodes

The printed Zn-CB-based anode was dried at 70 °C for 2 h to promote the solidification of the PVA binder. Afterward, the Zn-CB electrodes were treated in acetic acid (1%) to weld the Zn spheres and remove the residual oxidized layer of ZnO on the Zn spheres. Capillary Gr electrodes were thermally annealed in a tubular furnace at 350 °C for 30 min with argon gas flow (∼0.5 mbar) to carbonize the carboxymethyl cellulose binder.

### Electrochemical Deposition of MnO_2_ on Printed Gr Electrode

Manganese oxide (MnO_2_) was grown on conductive substrates and printed on 3D Gr scaffolds via a potentiostatic electrodeposition process. In a typical electrodeposition process, a three-electrode cell was set up in an aqueous electrolyte containing 0.1 M manganese­(II) acetate (Mn­(CH_3_COO)_2_, Alfa Aesar) and 0.1 M sodium sulfate (Na_2_SO_4_, Sigma-Aldrich) with a printed graphene scaffold as the working electrode. A graphite foil counter electrode and a conventional Ag/AgCl electrode as the reference electrode were used for the electrodeposition of MnO_2_. A linear sweep voltammetry test (Figure S7) was performed to adjust the electrodeposition potential of MnO_2_ on the 3D Gr electrode. Accordingly, an oxidative electrodeposition potential of ∼0.7 V was applied with different deposition times (15–75 min), which results in the formation of a MnO_2_ layer with a mass loading of ∼10 ± 0.1 mg/cm^2^ deposited on the Gr electrode. After the electrodeposition, the structures were rinsed with deionized water and dried on a hot plate at 60 °C.

### Fabrication and Characterization of Electrochemical Cells

Pouch-type symmetric cells and full cells were used to evaluate the cycling stability and the capacity of Zn-CB-based electrodes. Glassy microfiber separators (Φ16 mm, Whatman, Sigma-Aldrich) were employed to avoid short circuits between the electrodes. An aqueous 2 M ZnSO_4_ solution was used as a liquid electrolyte for symmetric cell measurements, and a prepared ZnSO_4_-silica colloid suspension (2 M ZnSO_4_ in a 5 wt % fumed silica) was used as a gel electrolyte in full cell measurements. Chronopotentiometry (CP) tests were performed on symmetric cells at current densities of 1 to 10 mA cm^–2^ to determine the plating-stripping overpotentials and stability of the Zn-CB anodes. The Coulombic efficiency (measured at different current densities of 0.5–10 mA cm^–2^) of the Zn-CB anodes was measured using asymmetric cells fabricated with copper foil as the cathode at different current densities. Tafel tests (corrosion measurements) were also conducted on printed anodes in a three-electrode system, using Zn or Zn-CB as the working electrode, Ag/AgCl as the reference electrode, and Pt as the counter electrode in a 1 M sodium sulfate (NaSO_4_, Alfa-Aesar) electrolyte, which was acidified to pH 5 to emulate the acidic condition of aqueous zinc sulfate employed in real cells. In situ mass spectroscopy was carried out to visualize the hydrogen evolution of the Zn-CB electrodes using ZnSO_4_ electrolyte. The full cell-based on zinc-ion batteries were assembled using woodpile-shaped 3D anode (Zn-CB) and electrochemical deposited cathodes (MnO_2_-Graphene) with a glassy fiber paper layer between them. An aqueous solution of 2 M ZnSO_4_ + 0.2 M MnSO_4_ was used as the electrolyte. Interdigitated Zn-ion batteries were also tested by printing a MnO_2_-graphene cathode and a Zn-CB anode on a gold (Au) interdigitated current collector. The prepared colloidal electrolyte was printed between the fingers of the interdigitated anode/cathode to design a fully printed microbattery. After the printing process, the interdigitated devices were wrapped with a parafilm foil or epoxy resin to prevent the evaporation of the electrolyte. The electrochemical tests including cycling voltammetry (CV), galvanic charge–discharge (GCD), and electrochemical impedance spectroscopy (EIS) measurements were performed on a multichannel VMP-3 workstation (Bio-Logic Science Instruments) and on a Gamry Interface 1000 potentiostat/galvanostat.

### Physical and Structural Characterization

Scanning electron microscopy (SEM, Zeiss Auriga) was employed to observe the surface properties of the electrodes, and the images were recorded by top view and the cross-sectional view. Transmission electron microscopy (TEM, JEOL JEM-200CX) was used to image the morphology and crystalline properties of MnO_2_. The sample was prepared using the following process: (1) MnO_2_ was dispersed in ethanol in a vial and sonicated for a few minutes; (2) using a micropipette, a drop of the prepared liquid was placed on the Cu grid composed of holey carbon support film and dried; (3) the sample was further dried on a hot plate at 60 °C until the solvent was completely evaporated. X-ray diffraction (XRD, Bruker D2 Phaser diffractometer with Cu Kα radiation of λ = 1.5406 Å) analysis was performed on pre- and postcycled Zn- and Zn-CB electrodes to investigate the formation of byproducts. X-ray computed tomography (XCT) was employed to visualize the internal structure of the printed electrodes and to obtain quantitative information on the size, distribution, and morphology of their features. The XCT measurements were performed on a Zeiss Xradia 620 Versa with a field of view of 961 μm. The radiographic projections were acquired in attenuation contrast, with a 0.48 μm voxel resolution. X-ray photoelectron spectroscopy (XPS, Thermo Fisher Kα- system with an Al Kα X-ray source) was used to study the surface composition and the chemical environment of the elements in MnO_2_. XPS characterization were performed on MnO_2_ directly grown on a graphite foil via electrodeposition, forming a compact layer of ∼100 μm in thickness. The acquired spectra were analyzed by using the Avantage software package. The mechanical properties of the printed structures were determined via compression tests performed on a universal testing machine. The mechanical tests were performed on a Zwick/Roell Z010 universal testing machine equipped with stainless steel compression plates. Compression tests were performed at a constant displacement rate of ∼0.5–1 mm min^–1^ until break on freestanding woodpile structures (∼1 cm^2^ footprint area). A preload of 10 N was applied before the measurements. The electrical conductivity of the inks was determined with four probe measurements. The inks were coated on glass substrates and dried overnight at room temperature. Four equally spaced contacts were made on the films by using conductive silver paint (RS Components). A current ramp was applied between the two outer contacts, and the potential fall between the inner contacts was measured using a Gamry Interface 1000 potentiostat.

## Supplementary Material



## Notes

Particle distribution of Zn powder; *I*–*V* plots of Zn inks; swelling ratio for different polymeric binders; oscillatory frequency sweep tests for the zinc inks (with and without carbon black); flow ramp tests; stress–strain curves for 3D printed zinc electrodes; plating/stripping electrochemical tests of Zn//Cu cells; plating/stripping overpotential of the 3D printed zinc-CB-based symmetric cells; EIS analysis of the symmetric cells; Tafel polarization plots; LSV curves of MnO_2_ deposition; SEM/EDX analysis of MnO_2_ deposition on printed graphene; rheology of the printable electrolytes; two-electrode system-based conductivity test of the printable electrolytes; CV curves of the printed battery and power law analysis; XRD profiles of the printed cathode after cycling tests; GCD and rate tests of the large area microbattery; CV curves of the interdigitated microbattery; performance comparison tables (PDF)
