# Supplementary material for: A Reversible Zinc-Ion Microbattery from a Printed Gel-Electrolyte and a Carbon–Zinc Formulation
Source: ACS Appl Mater Interfaces. 2025 Jun 23;17(26):38215–24. doi: 10.1021/acsami.5c05599 (PMC12232270; doi:10.1021/acsami.5c05599)
Supplement: Supplementary file 1 [file am5c05599_si_001.pdf]

Supporting Information

A reversible zinc-ion microbattery from a printed gel-electrolyte and a carbon-zinc formulation

Stefano Tagliaferri,[a‡] Nagaraju Goli,[a‡] Maria S. Sokolikova,[a] Haoyu Bai,[a] Caiwu Liang,[a] Ifan E.L. Stephens,[a] Cecilia Mattevi[a]*

[a] Department of Materials, Imperial College London, London SW7 2AZ, United Kingdom

*Corresponding author: c.mattevi@imperial.ac.uk

‡ S.T and N.G are equally contributed to this work

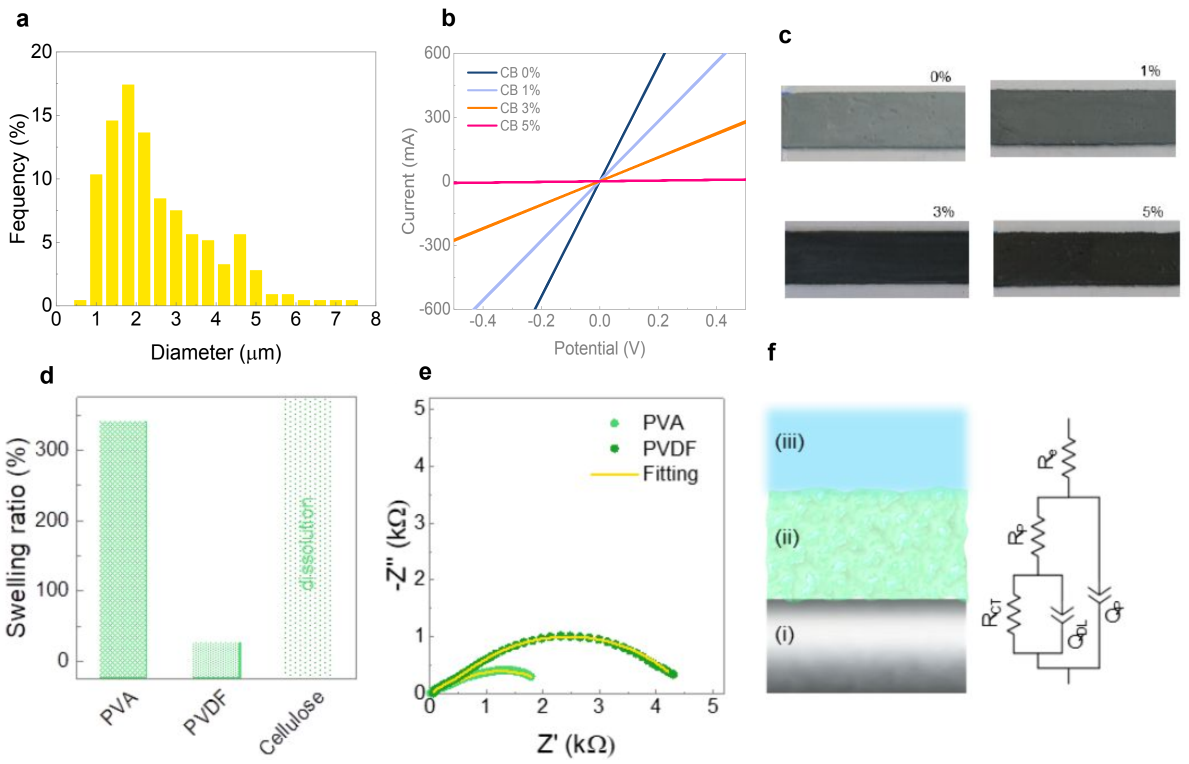

Figure S1. (a) Particle size distribution of zinc powder, (b) current potential curves for the zinc structures with different concentrations of carbon black and (c) Photographs of the thin-film zinc (with or without carbon black) electrodes used to study the conductivity measurements. d) Swelling ratio for different polymeric binders; (e) Nyquist plot for the symmetric polymer-coated zinc cell, with a PVA and PVDF coating, respectively; (f) schematic showing the polymer coated electrode and the equivalent circuit used to model the electrode; the resistive element RE describes the high-frequency resistance of the electrolyte (iii), the RP–QP parallel describes the resistance to transport and the capacitance of the polymeric coating (ii), whereas the parallel RCT–QDL describes the capacitance and charge transfer resistance at the interface with the zinc electrode (i). In our work, we choose PVA binder for the zinc ink formulation because of their hydrophilicity and sustainability versus polyvinylidene fluoride which is a widely used binder for tape-casted battery electrodes.[1] However, it is highly hydrophobic, and it is unable to swell in aqueous electrolytes, presenting an electrolyte uptake lower than 30% (Figure S1d) which leads to high interface resistance (Figure Sd).

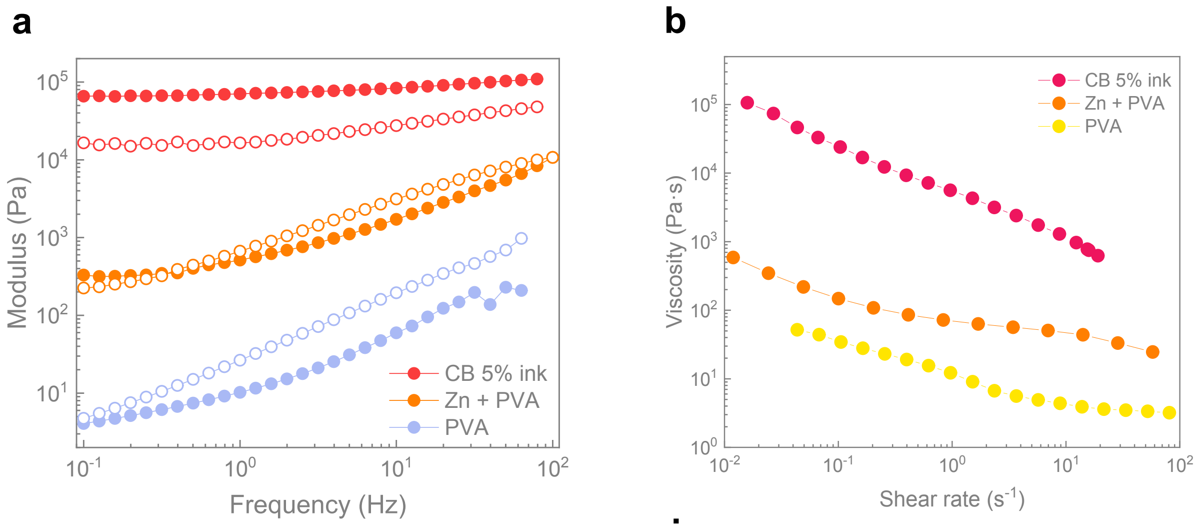

Figure S2: (a) Oscillatory frequency sweep tests for the zinc inks (with and without carbon black) and pristine PVA ink (closed circles represent G′, open circles G″). (b) flow ramp tests on the corresponding inks at increasing total weight fraction of solid material and corresponding power law fitting.

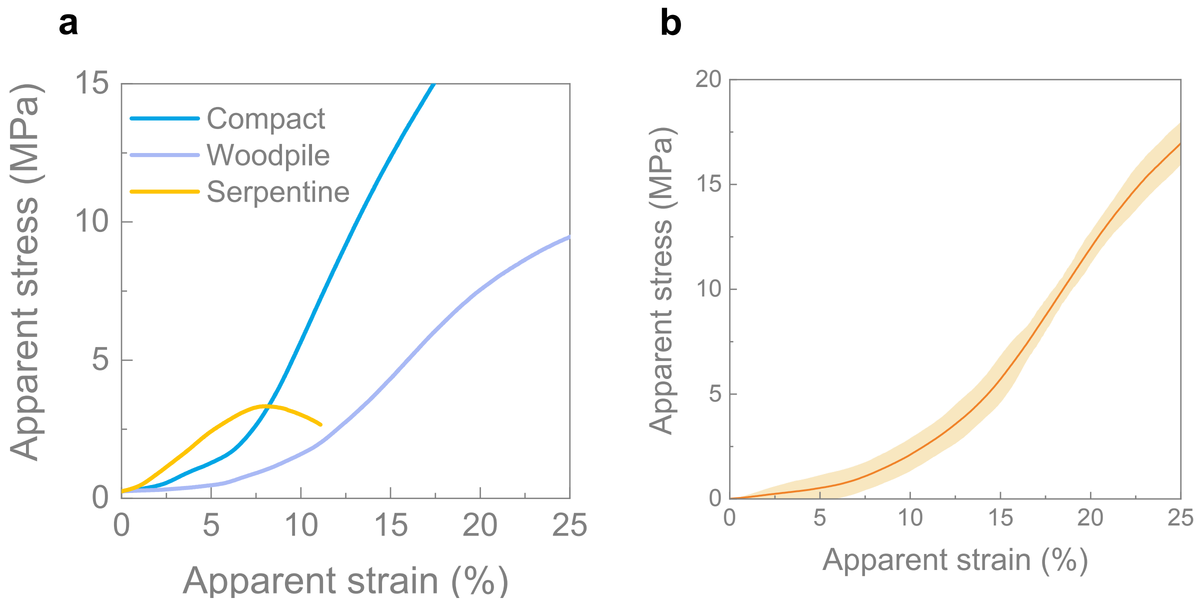

Figure S3. (a) Comparison of compression stress-strain curves for 3D printed zinc electrodes with different geometries; (b) Compression stress-strain curve on a cylindrical woodpile zinc electrode (5% carbon black), showing a progressive increase in the sample stiffness caused by densification (marked with orange dotted lines).

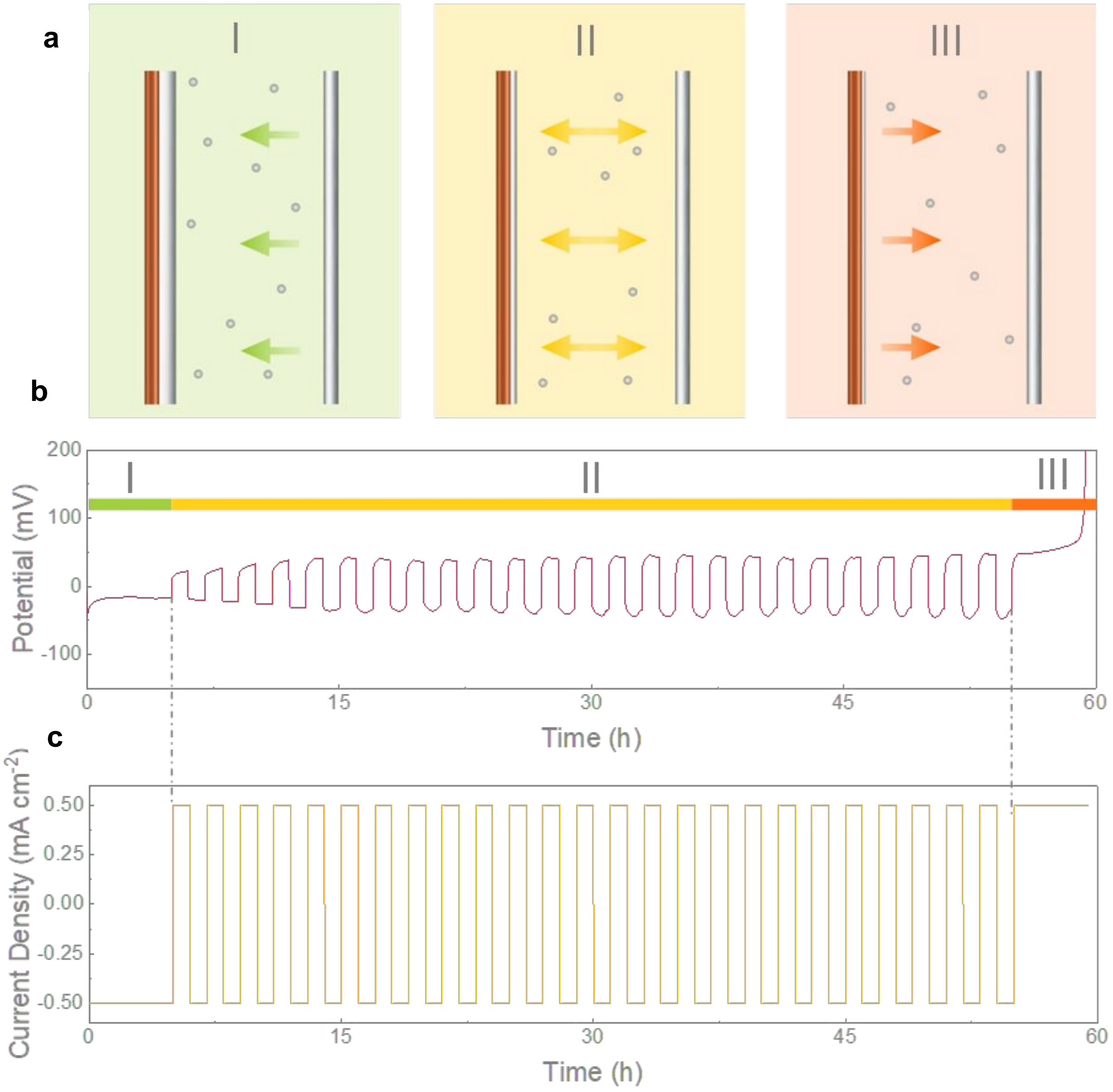

Figure S4. (a) Schematic showing the three steps of the reservoir method to measure coulombic efficiency: (1) formation of the reservoir, (2) cycling, (3) stripping of the residual reservoir; (b) corresponding potential profile in a typical test. b) potential profile for the coulombic efficiency tests with the reservoir method, presenting three regions: formation of the zinc reservoir on the copper counter electrode (1), cycling between the reservoir and the 3D printed electrode (2), stripping of the residual zinc in the reservoir (3). c) Constant current density is used to plate and strip the zinc from the asymmetric cell.

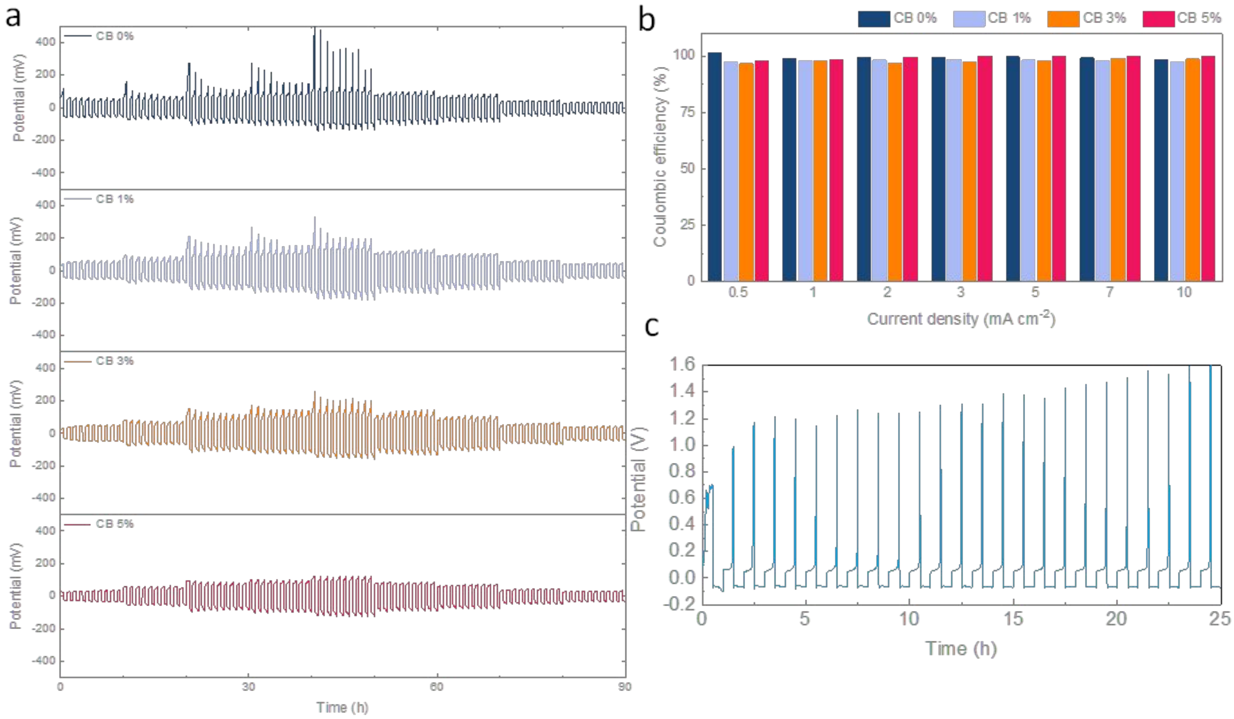

Figure S5. a) 2D demonstration showing the comparison of the plating-stripping overpotential of the 3D printed zinc-CB-based symmetric cells at increasing current density values, (b) comparison of the Coulombic efficiency of the 3D printed electrodes with different carbon black content. c) Galvanostatic cycling of pristine Zn-based symmetric cells, showing the high polarization behavior.

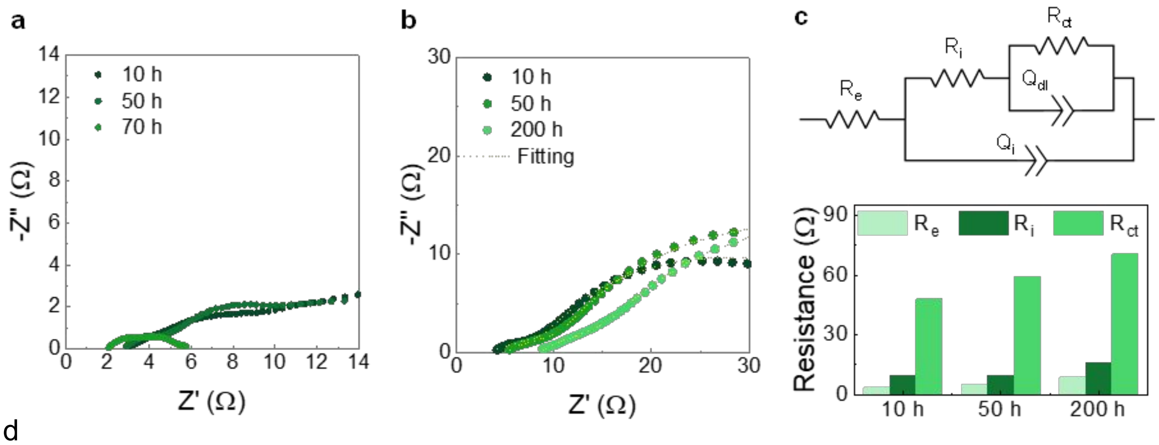

d

	10 h	50 h	200 h
R_E (Ω)	3.5	5.0	8.6
R_i (Ω)	9.7	9.4	16.2
Q_i (mF s^{n-1})	19.8	16.2	21.5
n_i	0.32	0.35	0.35
R_{CT} (Ω)	48.0	59.1	70.6
Q_{DL} (mF s^{n-1})	12.9	14.9	10.4
n_{DL}	0.75	0.74	0.77

Figure S6: (a) EIS on the symmetric zinc foil cell at different cycling times; (b) EIS on the symmetric 3D printed cell (zinc-CB 5% ink) at different cycling times; (c) equivalent circuit used to fit the impedance data (top) and histogram reporting the variation in the model resistances over cycling (bottom) for the symmetric 3D printed electrodes (zinc-CB 5% ink); (d) EIS fitting parameters for the symmetric 3D printed cell (zinc-CB 5% ink) after different cycling times.

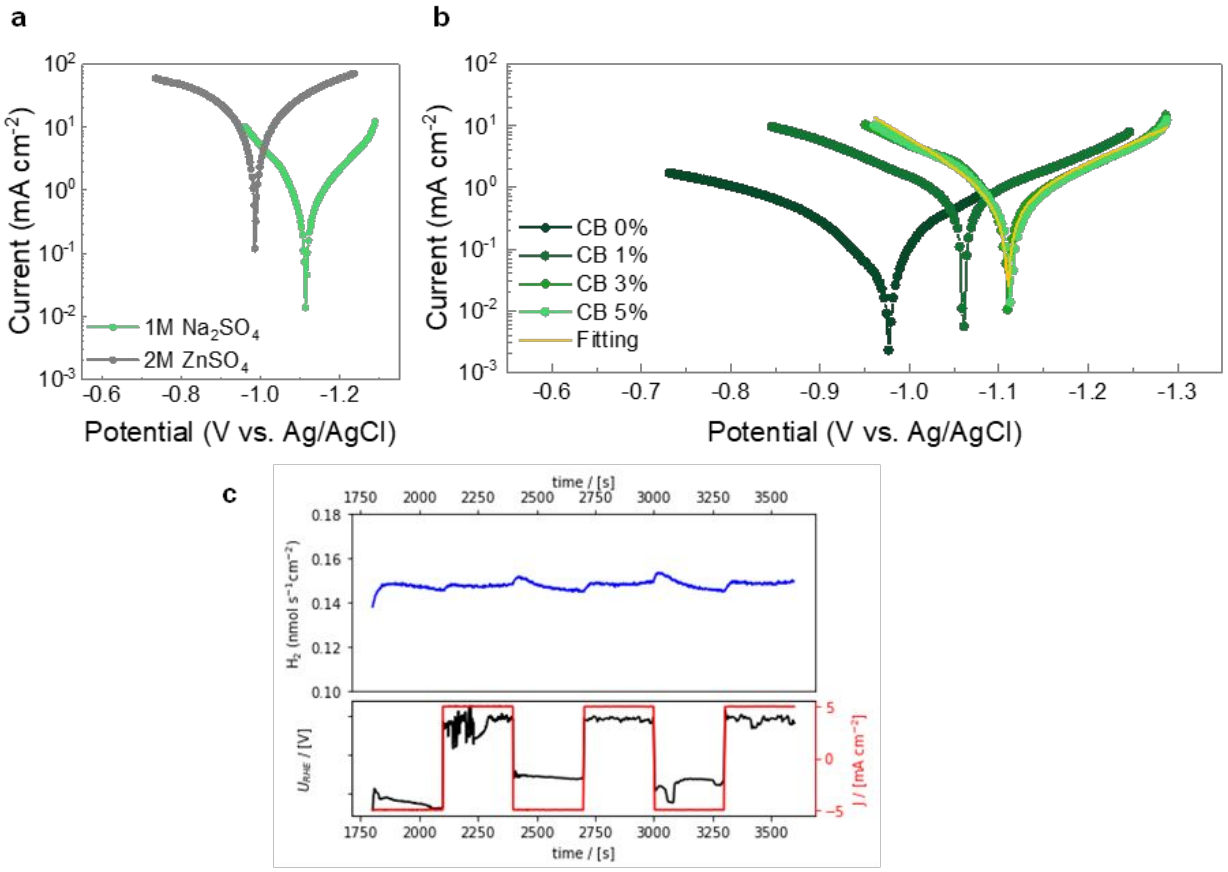

d

	CB 5%	CB 3%	CB 1%	CB 0%
βa (mV/decade)	117	87	140	98
βc (mV/decade)	161	165	164	53
i_{corr} (mA)	731	698	634	45
E_{corr} (V)	-1.11	-1.11	-1.06	-0.98

Figure S7: (a) Tafel polarization curves on the zinc-CB 5% ink in different electrolytes (scan rate 5 mV s^{-1}). (b) comparison of the Tafel polarization curves of the zinc inks with different content of carbon black in a 1M Na_2SO_4 electrolyte (acidified to pH 5) and Tafel fitting. (c) *in-situ* mass spectrometry experiments on the 5% carbon black ink, measuring the rate of hydrogen evolution during galvanostatic charge discharge. d) Tafel plot fitting parameters.

Corrosion analysis using Linear sweep voltammetry (LSV): The corrosion of the zinc inks at different carbon black contents was studied in a 1 M Na_2SO_4 electrolyte, acidified to pH 5 to emulate the acidic condition of aqueous zinc sulfate employed in real cells (Figure S7a,b). Compared to linear sweep voltammograms in a $ZnSO_4$ electrolyte, the curve in Na_2SO_4 shifts to

lower currents and a higher corrosion potential (Figure S7-a), because of the different redox processes being probed in the two electrolytes. The higher current density observed in concentrated $ZnSO_4$is associated with the deposition of the abundant zinc ions in the proximity of the electrode and does not provide information on the rate of hydrogen evolution. In the sodium sulfate electrolyte, the corrosion current progressively in-creases with the amount of carbon black in the ink (Figure S7b and S7d), even though the recent literature has reported that a carbon coating can have a protective action against corrosion.[2, 3] The increase in the corrosion current with carbon black content can be attributed to two factors: (1) as observed from SEM images (Figure 1 in the main manuscript), carbon black does not form a continuous layer protecting the zinc particles, but it mainly concentrates at the junction between particles and in the voids of the network; (2) the presence of a conductive network of carbon black enhances the number of zinc particles that are electrically connected to the current collector, increasing the electrochemically active surface area of the sample. Therefore, the 5% carbon black electrode presents the highest corrosion current (~ 731 μA cm^{-2}), corresponding to a corrosion rate of ~ 3.8 nmol s-1 cm^{-2}. Although the tests in 1 M Na_2SO_4 are useful to compare the corrosion behavior of the different inks, they cannot deliver quantitative information on the rate of zinc corrosion in a zinc sulfate electrolyte.

S.no	Anodes	Voltage hysteresis (mV)	Current density (mA cm^{-2})	SI. Ref.
1	This work	32.2	1.0	Zinc-CB 5%
2	This work	53.8	2.0	
3	This work	82.9	5.0	
4	This work	97.35	7.0	
5	This work	122.55	10	
6	MXenes@Zn	44.2	0.2	[4]
7	MXenes@Zn	117.3	5.0	[4]
8	TiO2@Zn	58.9	1.0	[5]
9	CNTs@Zn	78.8	0.5	[6]
10	Kaolin@Zn	100.1	1.8	[7]
11	MoS_2@Zn	117.9	2.5	[8]
12	ZrO2@Zn	151.3	5.0	[9]
13	rGO@Zn	201.1	2.0	[10]

Table S1. Comparison of the voltage hysteresis for the plating and stripping of zinc-CB 5% electrodeswith other zinc anodes recently reported in the literature.

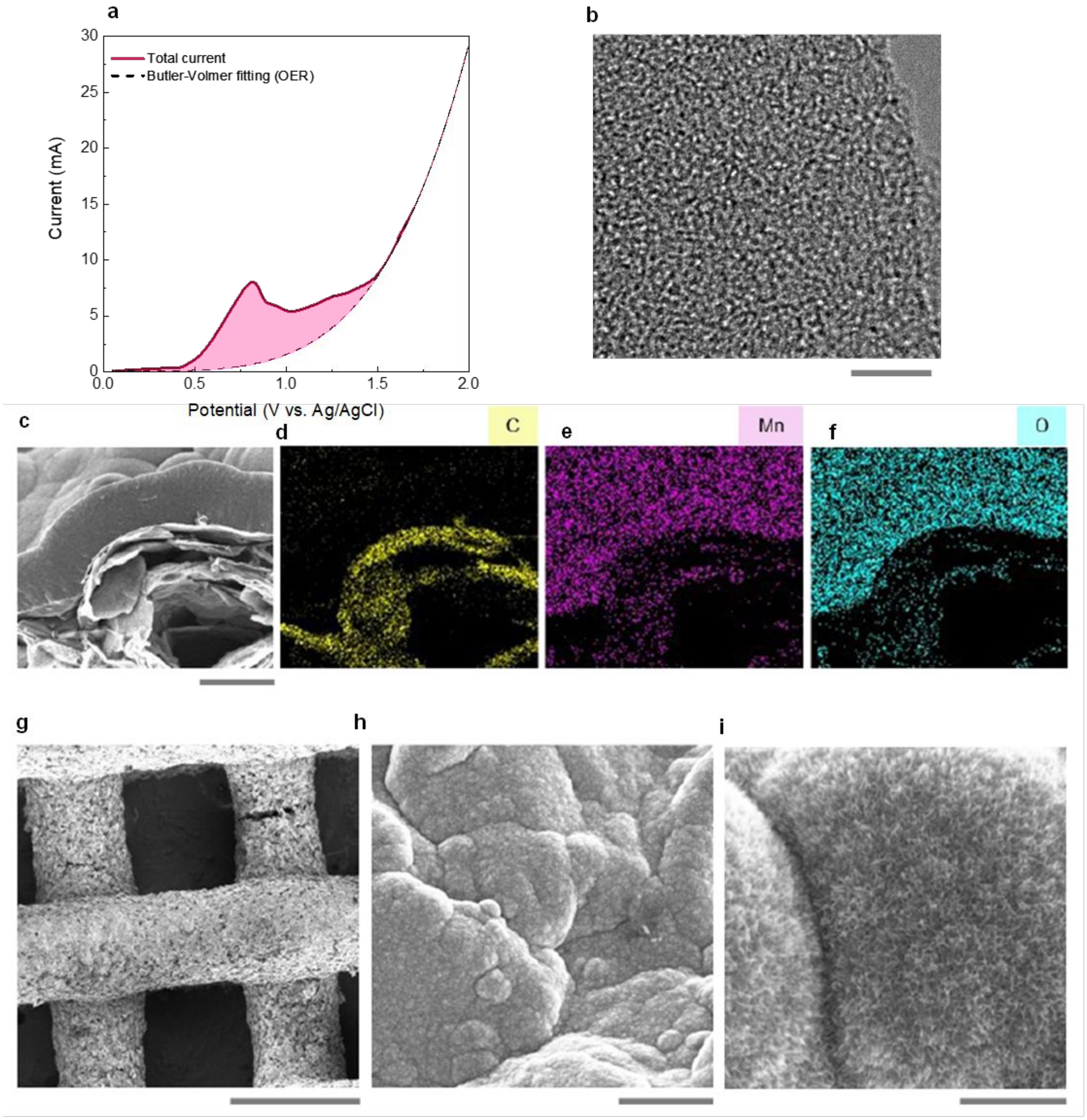

Figure S8. (a) Linear sweep voltammetry analysis used to optimize the electrochemical deposition potential of MnO_2 deposited on a graphene electrode in 0.1M $Mn(CH_3COO)_2$ and 0.1 M Na_2SO_4 solution; (b) high resolution TEM on the MnO_2 sheets grown on graphite nanoplatelets, revealing no obvious crystalline regions (scale bar 5 nm); (c) SEM cross-sectional image showing a thick layer of manganese oxide growing on the external surface of a graphene electrode, and (d-f) corresponding EDS mapping (scale bar 5 µm); (g-i) Low- and magnified SEM images on the external surface of a 3D printed graphene electrode after deposition with MnO_2 (scale bars 500 µm, 5µm and 1µm, respectively).

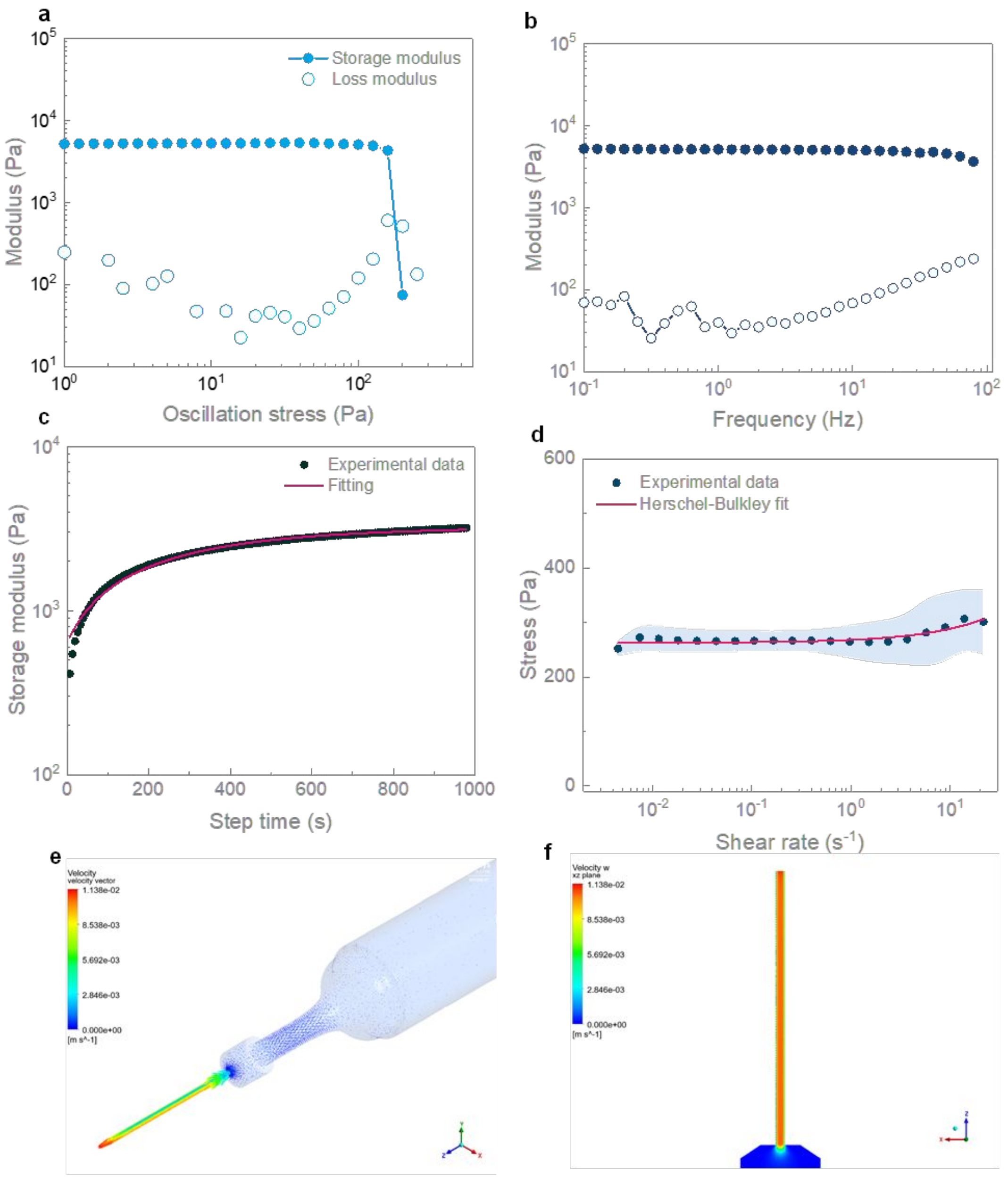

Figure S9. (a) oscillatory frequency sweep on the silica gel electrolyte within the LVE (G'close circles, G''open circles); (b) oscillatory amplitude sweep on the silica gel electrolyte at an oscillation frequency of 1 Hz (G'close circles, G''open circles); c) storage modulus recovery and exponential fitting of the 3ITTs in panel (d) flow ramp and Herschel-Bulkley fitting for the gel electrolyte. (e) Velocity profile of the gel electrolyte in the extrusion cartridge and nozzle determined with CFD simulations (extrusion speed 6 mm s^{-1}); (f) velocity profile (x-and z-component of the velocity vector) inside and in the proximity of the extrusion nozzle.

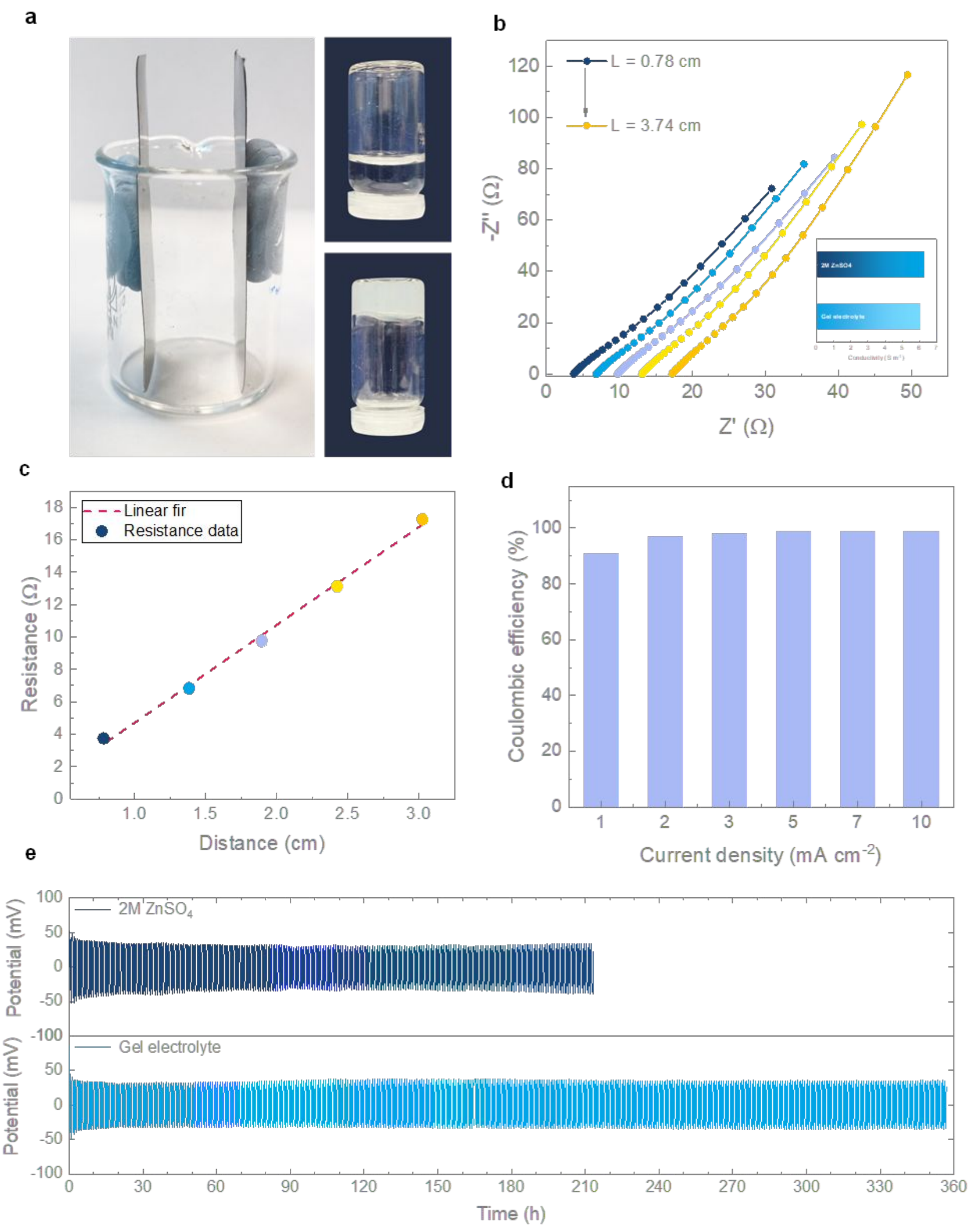

Figure S10. (a) Two-electrode system (electrodes are stainless steel foils) used to measure the EIS conductivity of prepared electrolytes; photographs on the right showing vial inversion tests on a 2M $ZnSO_4$ solution before and after the addition of fumed silica (5 wt%). (b) Electrochemical impedance spectroscopy on the gel electrolyte at increasing distance between the stainless-steel electrodes; inset of (b) is the conductivity comparison between conventional 2M $ZnSO_4$ electrolyte and as-prepared gel-electrolyte. (c) resistance values of the gel electrolyte (determined from EIS)

as a function of the distance between the stainless-steel electrodes; (d) coulombic efficiency of the gel electrolyte at different current densities; (e) galvanostatic plating-stripping tests on a symmetric zinc electrodes (foils) cell with 2M $ZnSO_4$ electrolyte and as-prepared gel-electrolyte.

S.No	Ionic Conductivity ($S\ m^{-1}$)	Transference Number	SI. References
1	6.043	0.362	This work
2	3.2	0.656	[11]
3	0.215	0.93	[12]
4	0.298	0.59	[13]
5	1.886	0.8	[14]
6	2.01	0.4	[15]
7	3.29	0.84	[16]
8	1.79	0.41	[17]
9	1.91	0.23	[18]

Table S2. comparison of the transference number and ionic conductivity of the gel electrolyte with zinc-ion gel electrolytes recently reported in the literature.

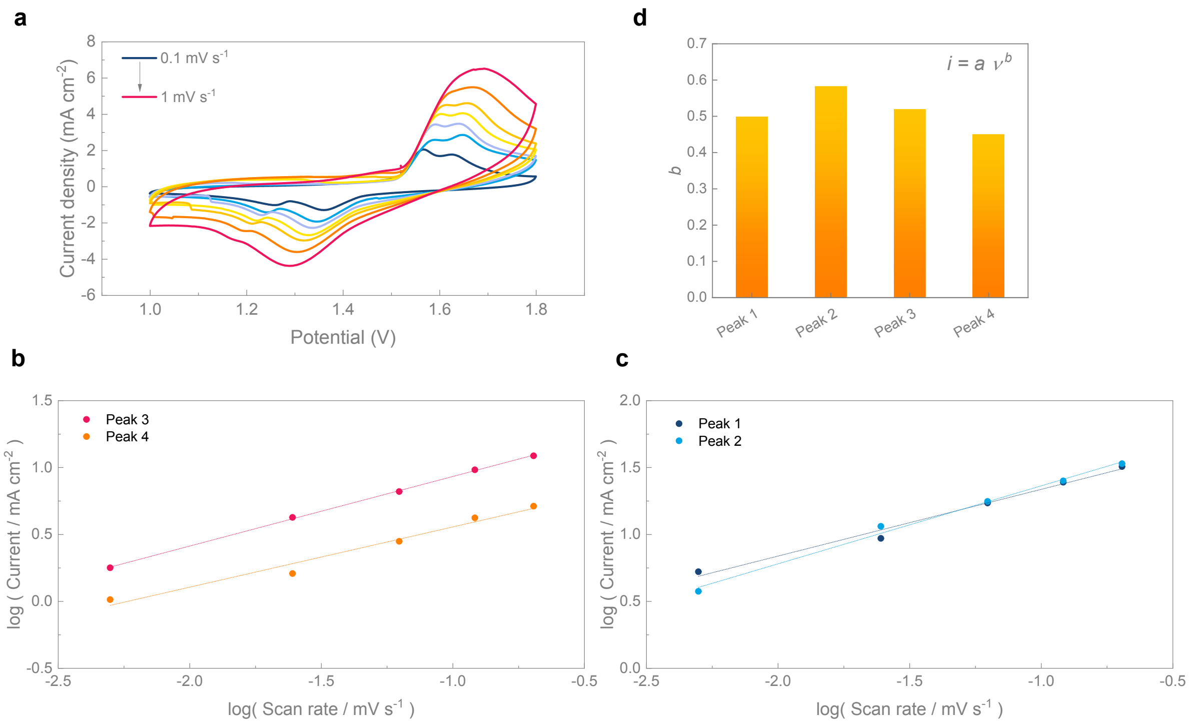

Figure S11. (a) CV curves of the woodpile shaped printed ZIB at different scan rates. (b)-(d) Power law fittings of peak current values vs. scan rates and corresponding slope (*b*-values) values of the redox peaks.

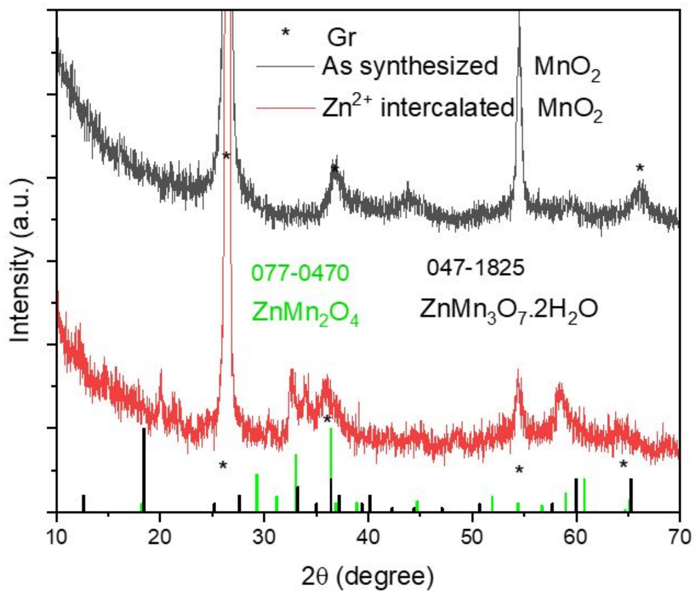

Figure S12. XRD spectra of electrochemically deposited MnO_2 on 3D printed conductive graphene (Gr) and Zn^{2+} intercalated MnO_2.

The X-ray diffraction (XRD) pattern in Figure S12 shows the structural evolution of electrochemically deposited MnO_2 on pristine graphene upon cycling in presence of 2M $ZnSO_4$ at 1.8V. Initially, a predominantly amorphous structure on MnO_2 emerged from the XRD pattern of the as-synthesized MnO_2. However, after the intercalation of Zn^{2+} ions, the pattern reveals the formation of $ZnMn_2O_4$, characterized by sharp and distinct peaks in a spinel like crystal structure.

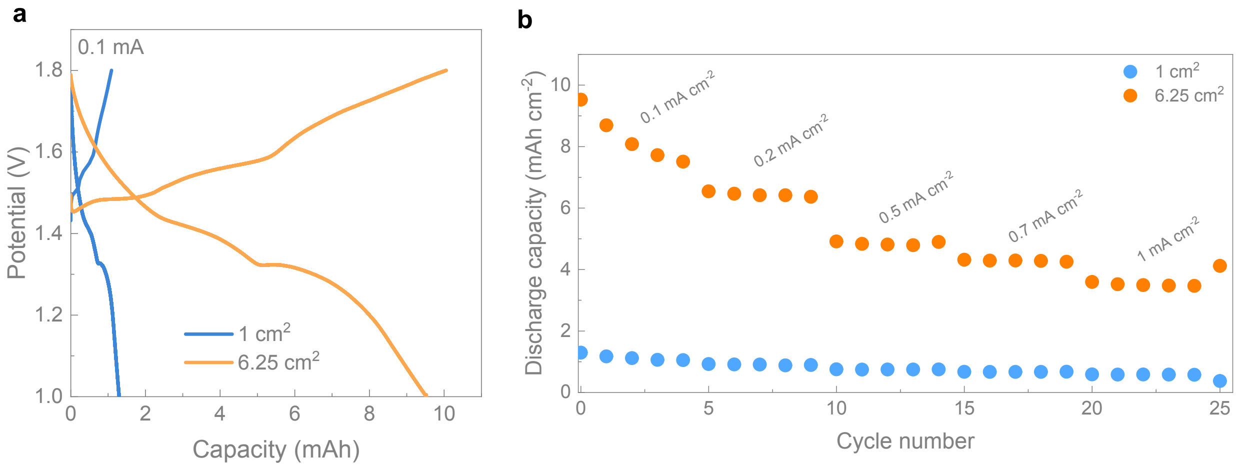

Figure S13. Comparative (a) galvanostatic-charge discharge profiles on the printed ZIBs cells with different footprint areas and (b) rate performance of the corresponding cells with different footprint areas.

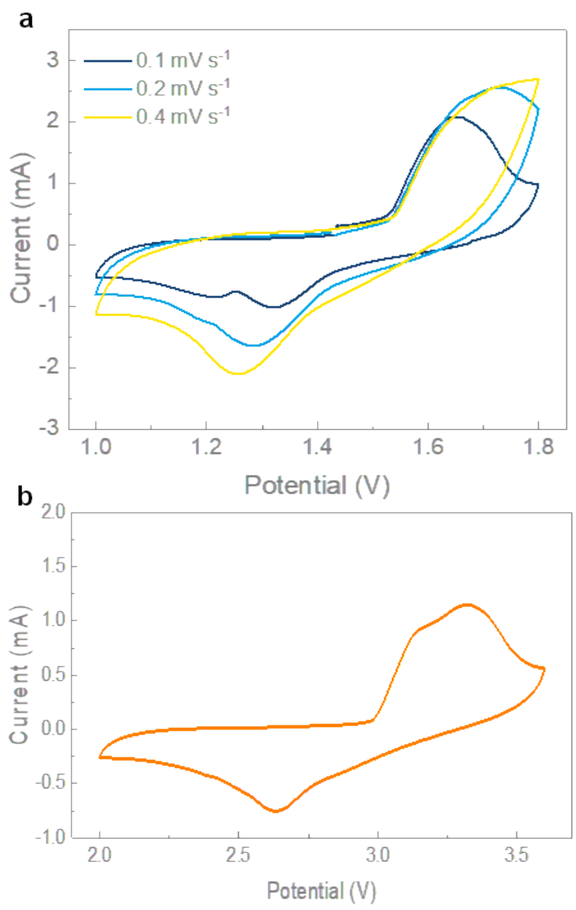

Figure S14: (a) cyclic voltammograms on a fully printed interdigitated battery encapsulated in epoxy resin; (b) cyclic voltammogram on the series of two interdigitated batteries.

S.No.	Printed batteries	Areal capacity (mAh cm^{-2})	Current (mA/cm^2)	SI. References
1	3DP Zn/CNT@MnO_2	0.063	0.4	[19]
2	Textile Zn/Ni-Co	0.076	--	[20]
3	ZnO/NiO	0.39	0.5	[21]
4	3DP Na-Ion	1.26	0.42	[22]
5	3D Zn/MnO_2 (gel)	0.305	0.37	[24]
6	Zn|PASHE|AC	0.1097	0.6	[23]
7	3D Zn/MnO_2	0.51	0.057	[24]
8	IL Zn/MnO_2	0.98	--	[25]
9	Zn-Ag	1.245	0.2	[26]
10	3D zinc-CB5%//MnO_2	1.3	0.1	This work

Table S3. comparison of the areal capacity of our 3D printed ZIBs with previously reported printed batteries.

SI References

1. Sudhakaran, S.; Bijoy, T. K. A Comprehensive Review of Current and Emerging Binder Technologies for Energy Storage Applications. *ACS Applied Energy Materials* **2023**, *6* (23), 11773-11794. DOI: 10.1021/acsaem.3c02218.
2. Wu, C.; Xie, K.; Ren, K.; Yang, S.; Wang, Q. Dendrite-free Zn anodes enabled by functional nitrogen-doped carbon protective layers for aqueous zinc-ion batteries. *Dalton Transactions* **2020**, *49* (48), 17629-17634, 10.1039/D0DT03459B. DOI: 10.1039/D0DT03459B.
3. Li, Z.; Wu, L.; Dong, S.; Xu, T.; Li, S.; An, Y.; Jiang, J.; Zhang, X. Pencil Drawing Stable Interface for Reversible and Durable Aqueous Zinc-Ion Batteries. *Advanced Functional Materials* **2021**, *31* (4), 2006495. DOI: https://doi.org/10.1002/adfm.202006495.
4. Zhang, N.; Huang, S.; Yuan, Z.; Zhu, J.; Zhao, Z.; Niu, Z. Direct Self-Assembly of MXene on Zn Anodes for Dendrite-Free Aqueous Zinc-Ion Batteries. *Angewandte Chemie International Edition* **2021**, *60* (6), 2861-2865. DOI: https://doi.org/10.1002/anie.202012322.
5. Zhao, K.; Wang, C.; Yu, Y.; Yan, M.; Wei, Q.; He, P.; Dong, Y.; Zhang, Z.; Wang, X.; Mai, L. Ultrathin Surface Coating Enables Stabilized Zinc Metal Anode. *Advanced Materials Interfaces* **2018**, *5* (16), 1800848. DOI: https://doi.org/10.1002/admi.201800848.
6. Li, M.; He, Q.; Li, Z.; Li, Q.; Zhang, Y.; Meng, J.; Liu, X.; Li, S.; Wu, B.; Chen, L.; et al. A Novel Dendrite-Free Mn2+/Zn2+ Hybrid Battery with 2.3 V Voltage Window and 11000-Cycle Lifespan. *Advanced Energy Materials* **2019**, *9* (29), 1901469. DOI: https://doi.org/10.1002/aenm.201901469.
7. Deng, C.; Xie, X.; Han, J.; Tang, Y.; Gao, J.; Liu, C.; Shi, X.; Zhou, J.; Liang, S. A Sieve-Functional and Uniform-Porous Kaolin Layer toward Stable Zinc Metal Anode. *Advanced Functional Materials* **2020**, *30* (21), 2000599. DOI: https://doi.org/10.1002/adfm.202000599.
8. Bhoyate, S.; Mhin, S.; Jeon, J.-e.; Park, K.; Kim, J.; Choi, W. Stable and High-Energy-Density Zn-Ion Rechargeable Batteries Based on a MoS2-Coated Zn Anode. *ACS Applied Materials & Interfaces* **2020**, *12* (24), 27249-27257. DOI: 10.1021/acsami.0c06009.
9. Liang, P.; Yi, J.; Liu, X.; Wu, K.; Wang, Z.; Cui, J.; Liu, Y.; Wang, Y.; Xia, Y.; Zhang, J. Highly Reversible Zn Anode Enabled by Controllable Formation of Nucleation Sites for Zn-Based Batteries. *Advanced Functional Materials* **2020**, *30* (13), 1908528. DOI: https://doi.org/10.1002/adfm.201908528.
10. Shen, C.; Li, X.; Li, N.; Xie, K.; Wang, J.-g.; Liu, X.; Wei, B. Graphene-Boosted, High-Performance Aqueous Zn-Ion Battery. *ACS Applied Materials & Interfaces* **2018**, *10* (30), 25446-25453. DOI: 10.1021/acsami.8b07781.
11. Zhu, M.; Wang, X.; Tang, H.; Wang, J.; Hao, Q.; Liu, L.; Li, Y.; Zhang, K.; Schmidt, O. G. Antifreezing Hydrogel with High Zinc Reversibility for Flexible and Durable Aqueous Batteries by Cooperative Hydrated Cations. *Advanced Functional Materials* **2020**, *30* (6), 1907218. DOI: https://doi.org/10.1002/adfm.201907218.
12. Zhang, W.; Guo, F.; Mi, H.; Wu, Z.-S.; Ji, C.; Yang, C.; Qiu, J. Kinetics-Boosted Effect Enabled by Zwitterionic Hydrogel Electrolyte for Highly Reversible Zinc Anode in Zinc-Ion Hybrid Micro-Supercapacitors. *Advanced Energy Materials* **2022**, *12* (40), 2202219. DOI: https://doi.org/10.1002/aenm.202202219.
13. Cong, J.; Shen, X.; Wen, Z.; Wang, X.; Peng, L.; Zeng, J.; Zhao, J. Ultra-stable and highly reversible aqueous zinc metal anodes with high preferred orientation deposition achieved by a polyanionic hydrogel electrolyte. *Energy Storage Materials* **2021**, *35*, 586-594. DOI: https://doi.org/10.1016/j.ensm.2020.11.041.

14. Chan, C. Y.; Wang, Z.; Li, Y.; Yu, H.; Fei, B.; Xin, J. H. Single-Ion Conducting Double-Network Hydrogel Electrolytes for Long Cycling Zinc-Ion Batteries. *ACS Applied Materials & Interfaces* **2021**, *13* (26), 30594-30602. DOI: 10.1021/acsami.1c05941.
15. Leng, K.; Li, G.; Guo, J.; Zhang, X.; Wang, A.; Liu, X.; Luo, J. A Safe Polyzwitterionic Hydrogel Electrolyte for Long-Life Quasi-Solid State Zinc Metal Batteries. *Advanced Functional Materials* **2020**, *30* (23), 2001317. DOI: https://doi.org/10.1002/adfm.202001317.
16. Huang, S.; Hou, L.; Li, T.; Jiao, Y.; Wu, P. Antifreezing Hydrogel Electrolyte with Ternary Hydrogen Bonding for High-Performance Zinc-Ion Batteries. *Advanced Materials* **2022**, *34* (14), 2110140. DOI: https://doi.org/10.1002/adma.202110140.
17. Fu, C.; Wang, Y.; Lu, C.; Zhou, S.; He, Q.; Hu, Y.; Feng, M.; Wan, Y.; Lin, J.; Zhang, Y.; Pan, A. Modulation of hydrogel electrolyte enabling stable zinc metal anode. *Energy Storage Materials* **2022**, *51*, 588-598. DOI: https://doi.org/10.1016/j.ensm.2022.06.034.
18. Li, C.; Zhang, Q.; Sun, J.; Li, T.; E, S.; Zhu, Z.; He, B.; Zhou, Z.; Li, Q.; Yao, Y. High-Performance Quasi-Solid-State Flexible Aqueous Rechargeable Ag–Zn Battery Based on Metal–Organic Framework-Derived Ag Nanowires. *ACS Energy Letters* **2018**, *3* (11), 2761-2768. DOI: 10.1021/acsenergylett.8b01675.
19. Ren, Y.; Meng, F.; Zhang, S.; Ping, B.; Li, H.; Yin, B.; Ma, T. CNT@MnO_2 composite ink toward a flexible 3D printed micro-zinc-ion battery. Carbon Energy 2022, 4 (3), 446-457. DOI: https://doi.org/10.1002/cey2.177.
20. Huang, Y.; Ip, W. S.; Lau, Y. Y.; Sun, J.; Zeng, J.; Yeung, N. S. S.; Ng, W. S.; Li, H.; Pei, Z.; Xue, Q.; et al. Weavable, Conductive Yarn-Based NiCo//Zn Textile Battery with High Energy Density and Rate Capability. ACS Nano 2017, 11 (9), 8953-8961. DOI: 10.1021/acsnano.7b03322.
21. Liu, J.; Guan, C.; Zhou, C.; Fan, Z.; Ke, Q.; Zhang, G.; Liu, C.; Wang, J. A Flexible Quasi-Solid-State Nickel–Zinc Battery with High Energy and Power Densities Based on 3D Electrode Design. Advanced Materials 2016, 28 (39), 8732-8739. DOI: https://doi.org/10.1002/adma.201603038.
22. Ding, J.; Shen, K.; Du, Z.; Li, B.; Yang, S. 3D-Printed Hierarchical Porous Frameworks for Sodium Storage. ACS Applied Materials & Interfaces 2017, 9 (48), 41871-41877. DOI: 10.1021/acsami.7b12892.
23. W. Zhang et al., Kinetics-Boosted Effect Enabled by Zwitterionic Hydrogel Electrolyte for Highly Reversible Zinc Anode in Zinc-Ion Hybrid Micro-Supercapacitors. Advanced Energ materials. 2022, 12, 2202219
24. Liu, H.; Zhang, G.; Wang, L.; Zhang, X.; Zhao, Z.; Chen, F.; Song, L.; Duan, H. Engineering 3D Architecture Electrodes for High-Rate Aqueous Zn–Mn Microbatteries. ACS Applied Energy Materials 2021, 4 (9), 10414-10422. DOI: 10.1021/acsaem.1c02410.
25. Ho, C. C.; Evans, J. W.; Wright, P. K. Direct write dispenser printing of a zinc microbattery with an ionic liquid gel electrolyte. Journal of Micromechanics and Microengineering 2010, 20 (10), 104009. DOI: 10.1088/0960-1317/20/10/104009.
26. C. Li, et al.,.. High-performance quasi-solid-state flexible aqueous rechargeable ag-zn battery based on metal-organic framework-derived ag nanowires. *ACS Energy Lett* **3**, 2018. 11, 2761–2768
